# Shiga toxin increases intestinal transit to displace resident microbes and facilitate pathogen colonization

**DOI:** 10.1371/journal.ppat.1014104

**Published:** 2026-03-30

**Authors:** Max A. Odem, Sabona B. Simbassa, Cecilia Fadhel Alvarez, Oliyad Jeilu, Shelby R. Simar, Rachel Bosserman, Soumita Dutta, Walter Galdamez, Hossaena Ayele, Blake M. Hanson, Diana M. Proctor, Anne Marie Krachler

**Affiliations:** 1 Department of Microbiology and Molecular Genetics, The University of Texas Health Science Center at Houston, Houston, Texas, United States of America; 2 Microbiology and Infectious Diseases Program, University of Texas MD Anderson Cancer Center UTHealth Houston Graduate School of Biomedical Sciences, Houston, Texas, United States of America; 3 Center for Infectious Diseases, The UTHealth Houston School of Public Health at Houston, Houston, Texas, United States of America; INSERM U1220, FRANCE

## Abstract

Shiga toxin (Stx)-producing *Escherichia coli* (STEC) is a major cause of food-borne illnesses, and disease severity correlates with the production of Shiga toxins. While clinical symptoms such as bloody diarrhea and hemolytic uremic syndrome have been attributed to Stx, its contribution to bacterial fitness is not well understood. Here, we demonstrate that Stx2 enhances STEC colonization of the zebrafish gut by facilitating the partial displacement of gut resident microbes. Infection with Stx2-producing STEC strains or direct exposure of fish to purified Stx2 induces alterations in the zebrafish microbiome structure, impacting several bacterial phyla and genera, notably Pseudomonads. We show that Stx2 is sufficient to facilitate these changes by accelerating intestinal transit, leading to increased expulsion of select gut microbes, including resident *Pseudomonas* species. Additionally, prokinetic drug treatment causes similar changes in gut transit and expulsion of *Pseudomonas*. Collectively, these findings detail a novel mode of action of Stx2 on the host, and shed light on its contribution to bacterial fitness within the host intestine.

## Author summary

Shiga toxin (Stx)-producing *Escherichia coli* (STEC) are as major group of food-borne pathogens. STEC are known to cause severe gastrointestinal illnesses in humans, and its ability to produce Stx2 is a key factor in its virulence. In this study, we explored how Shiga toxin 2 (Stx2) influences the health of the zebra-fish intestine. Stx2, produced by STEC, boosts the colonization of *E. coli* in the gut, potentially affecting overall gut health. Additionally, exposure to Stx2 leads to changes in the composition of the intestinal microbiota, impacting both the number of microbial colonies and their distribution. Interestingly, Stx2 seems to play a role in displacing specific bacterial species, such as *Pseudomonas*, from the intestine. Furthermore, our study uncovers a novel function of Stx2 in speeding up the transit of substances through the zebrafish intestine, shedding light on a previously unknown aspect of its impact on gut dynamics.

## Introduction

Shiga toxin (Stx)-producing *Escherichia coli* (STEC) is a significant public health concern due to its ability to cause severe gastrointestinal disease, with Stx playing a pivotal role in virulence by disrupting host cell functions, inducing cell death, and contributing to systemic complications such as hemolytic uremic syndrome [1]. Stx achieves this by targeting ribosomal RNA in host cells, inhibiting protein synthesis, and leading to cell damage, particularly in the kidneys and vascular endothelium [2]. Stx also exerts profound neurotoxic effects, disrupting the central nervous system by inducing inflammation, oxidative stress and neuronal damage, which contribute to complications such as encephalopathy and seizures [3–5]. Enterohemorrhagic *E. coli* (EHEC) O157:H7 is one of the most prevalent STEC serotypes and can produce Shiga toxin variants Stx1, Stx2, or both toxins, with Stx2 considered more virulent and associated with more severe illness [6,7]. Although the relationship between Stx cellular intoxication and the above-mentioned host pathologies is well established, the mechanisms by which Stx enhances bacterial fitness remain poorly understood.

Several studies point toward a potential link between Stx, intestinal colonization, and the endogenous microbiota: Robinson et al. report that an EHEC strain lacking Stx2 exhibits reduced colonization in mice with a normal microbiota [8]. In contrast, Stx2 deficient strains are not colonization deficient in germ-free mice [9], and *ex vivo* colonization of colonic epithelium is also unaffected by Stx [10]. Although these studies indicate that Stx may play a crucial role in competing with the host microbiota, the specific mechanisms behind this competition remain unknown.

Here, we used a well-established zebrafish larval model of food-borne EHEC infection [11,12] to study how Stx impacts bacterial fitness during intestinal colonization. This model uses the protozoan *Paramecium caudatum* as a vehicle to deliver EHEC to the zebrafish intestine, where they colonize [13]. We show that in this model, EHEC causes drastic changes to the intestinal microbiota, and that Stx2 is both necessary and sufficient for these changes in microbiome structure. We further show that this dysbiosis is caused by increased bacterial shedding of key intestinal species, and that this displacement of bacteria is caused by Stx through changes in host intestinal transit rates. Our findings suggest a novel mechanism for Stx in bacterial colonization fitness, whereby the toxin’s effect on host physiology contributes to inter-bacterial competition.

## Results

### Shiga toxin 2 facilitates gut colonization by enterohemorrhagic Escherichia coli

**Fig 1:**
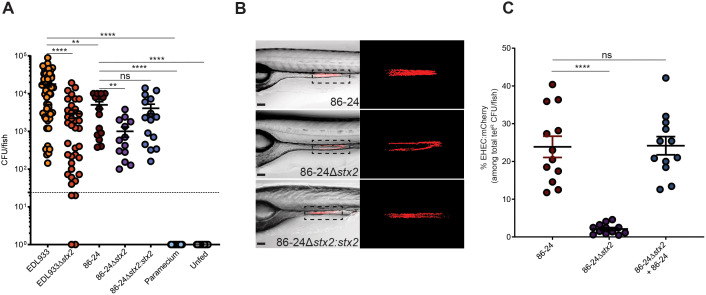
Shiga toxin 2 facilitates gut colonization by enterohemorrhagic *Escherichia coli.* **(A)** *E. coli* O157 CFUs (mauve colonies on EHEC CHROMagar) recovered from larval tissue homogenates at 7 dpf following food-borne infection of larvae for 24 hrs. Controls were larvae fed paramecia vehicle without EHEC, and unfed larvae. Data are individual fish (N = 50 for EDL933 strains, N = 15 for all other conditions), and means ± sem; **(B)** Images of 7 dpf larvae following 24 hr colonization with EHEC 86-24 wild type, ∆*stx*2, and ∆*stx*2*:stx*2 complement strains expressing mCherry. Right panels represent enlarged view of boxed area on the left. Representative images from N = 15 fish/group. **(C)** EHECmCherry+/tet^R^ as a fraction of total tet^R^ CFUs recovered from the gut of 7 dpf zebrafish following 24 hr infection with EHEC 86-24 WT:mcherry^tetR^, ∆*stx*2:mcherry^tetR^, or a 1:1 mixture of ∆*stx*2:mcherry^tetR^ and unmarked WT. Note that there are other tet^R^ bacteria present as part of the normal gut microbiota. Data are individual fish (N = 12/group), and means ± sem. Statistics: ANOVA and Dunnett’s multiple comparison test, ****p ≤ 0.0001, **p ≤ 0.01, ns (not significant) p ≥ 0.05; Samples with no detectable bacterial burden were plotted as 1 for visualization and statistical analysis. Dashed line = limit of detection.

Previous studies have reported conflicting results regarding the role of Stx in EHEC gut colonization [8,9]. To clarify this, we tested the contribution of Stx to colonization fitness using our food-borne infection model with larval zebrafish as a host. As wild type EHEC strains, we used the human-derived isolates EDL933, which produces both Stx1 and Stx2, and 86-24, which produces only Stx2. To assess the impact of these toxins on colonization fitness, we generated in-frame deletions of *stx*2 (*∆stx*2) and chromosomal complementation strains *(∆stx*2*:stx*2), (Table 1). Larval zebrafish at 7 days post fertilization (dpf) were colonized with both isolates and their derivatives using our previously developed food-borne infection model [11,13]. Gut colonization was determined 24 hours post-infection by dilution plating of homogenized zebrafish tissues on EHEC CHROMagar. Deletion of *stx*2 caused a 6-fold and 5-fold reduction in EHEC burden for EDL933 (Stx1 + 2) and 86-24 (Stx2 only) strains, respectively. Chromosomal complementation of *stx*2 restored wild type colonization levels, and as expected, no EHEC were present in the gut microbiota of unfed fish or fish fed paramecia vehicle alone (Fig 1A). Since previous studies indicated that Stx phage carriage affects bacterial persistence in the presence of grazing protozoa [19–21], we compared bacterial degradation by *P. caudatum* as described previously [13,22]. No significant differences were observed in bacterial persistence among wild type, *∆stx*2, and complemented strains within *paramecia* (Fig AA in S1 Appendix), indicating that fish received equal doses of wild type *E. coli*, deletion and complemented strains. Additionally, *in vitro* growth of EHEC strains was unaffected by *stx*2 deletion or complementation (Fig AB in S1 Appendix). We visualized the colonization pattern in EHEC infected fish using strains expressing mCherry (Fig 1B). Wild type, *stx*2 deletion, and complemented strains all colonized the larval midgut as previously described; however, bacterial burden was markedly reduced for the *stx*2 mutant. Next, we tested whether co-colonization with the wild type strain could restore colonization by the *stx*2 mutant. Fish were infected with either wild type or *stx*2 mutant individually, or with both strains in combination. While the *stx*2 mutant alone colonized approximately 20-fold less than the wild type strain, its fitness was restored by co-colonization with the wild type strain (Fig 1C). These results indicate that Stx2 contributes to EHEC colonization fitness, and that the reduced fitness of the *stx*2 mutant can be rescued by Stx2 produced by the wild type strain, consistent with findings in the conventional mouse model [8].

**Table 1:** Bacterial strains used in this study.

Strain	Description	Cytotoxicity	Source
EDL933 WT	EHEC O157:H7 wild type strain, contains both stx 1 and 2 phage	+++	[14]
EDL933 Δ*stx2*	Δstx2b::kan	+	This study
EDL933 Δ*stx2:stx2*	Δstx2b attB:stx2abTN7	+++	This study
86-24 WT	EHEC O157:H7 clinical isolate, contains stx2 phage, SmR, NaIR	+	[15]
86-24 Δ*stx2*	Δstx2b::kan	–	This study
86-24 Δ*stx2:stx2*	Δstx2b attB:stx2abTN7	+	This study
*E. coli* BL21 (DE3)	Protein expression strain; F − ompT hsdSB(rB − mB−) gal dcm (DE3)	NA	[16]
*E. coli* MG1655	Paramecium food strain	–	[17]
*Pseudomonas* zfem001	*Pseudomonas sediminis* isolate from the zebrafish gut	NA	[18]
*Pseudomonas* zfem002	*Pseudomonas japonica* isolate from the zebrafish gut	NA	[18]
*Pseudomonas* zfem003	*Pseudomonas otitidis* isolate from the zebrafish gut	NA	[18]
*Pseudomonas* zfem004	*Pseudomonas sichuanensis* isolate from the zebrafish gut	NA	[18]
*Pseudomonas* zfem005	*Pseudomonas tohonis* isolate from the zebrafish gut	NA	[18]

### Shiga toxin 2 facilitates partial displacement of the intestinal microbiota

**Fig 2:**
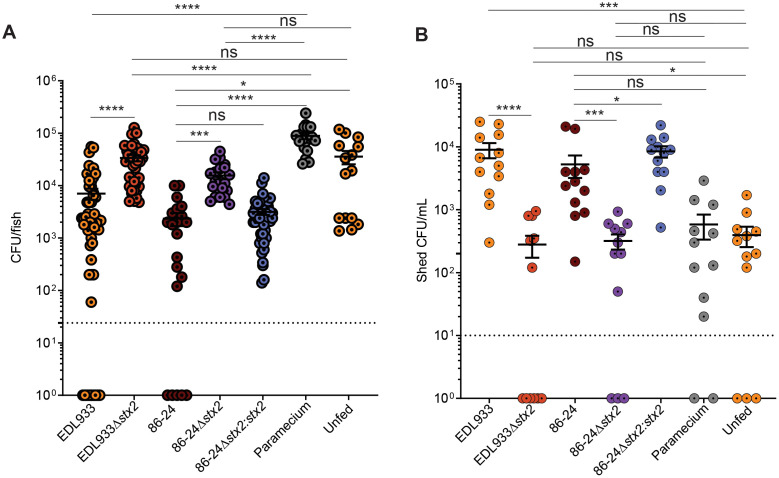
Shiga toxin 2 facilitates partial displacement of the intestinal microbiota. Non-EHEC CFUs (blue and white colonies on EHEC CHROMagar) recovered from larval tissue homogenates **(A)** or shed into the fish media **(B)** at 7 dpf following food-borne infection of larvae for 24 hrs. Controls were larvae fed paramecia vehicle without EHEC, and unfed larvae. Data are individual fish (N ≥ 15/condition) or media samples (N = 12), and means ± sem; Statistics: ANOVA and Dunnett’s multiple comparison test, ****p ≤ 0.0001, ***p ≤ 0.001, *p < 0.05, ns (not significant) p ≥ 0.05; Samples with no detectable bacterial burden were plotted as 1 for visualization and statistical analysis. Dashed line = limit of detection.

EHEC CHROMagar is a selective and differential chromogenic medium that allowed us to quantify EHEC (mauve colonies), and distinguish them from constituents of the normal zebrafish gut microbiota (non-EHEC coliforms, blue colonies, and other Gram-negatives, white colonies). In fish colonized with *stx*2 mutants the EHEC burden was consistently lower than in fish infected with wild type EHEC (Fig 1), while colonization with endogenous fish microbiota was consistently higher (Fig 2A). Uninfected fish showed the highest level of colonization by endogenous microbial constituents. At the same time, uninfected fish shed the lowest amount of endogenous microbiota into the fish medium (Fig 2B). Shedding of endogenous microbiota was significantly increased by the presence of Stx2 (Fig 2B, wild type vs *stx*2 mutants). The presence of Stx2 increased shedding, and constituents of the endogenous microbiota remained viable following shedding into the media, since shed bacteria replicated into colonies following CFU plating (Fig 2B). Furthermore, the Stx2 phage did not cause lysis of key members of the endogenous microbiota (Fig B in S1 Appendix), and the presence of purified Stx2 complex had no effect on the growth of these species (Fig C in S1 Appendix), [18,23]. Finally, we also tested whether the shed E3 media following Stx2 exposure of fish would contain any compounds that would accelerate growth of microbiota constituents, which would explain increased CFUs in shed media, but found no evidence of accelerated growth in shed media for any of the four strains isolated from the zebrafish microbiota (Fig D in S1 Appendix). Together, these results indicate that Stx2 contributes to colonization fitness by displacing endogenous microbial constituents from the gut through increased shedding by the host, but that the toxin has no direct antimicrobial effect.

### Shiga toxin 2 is sufficient to cause partial loss of the intestinal microbiota

**Fig 3:**
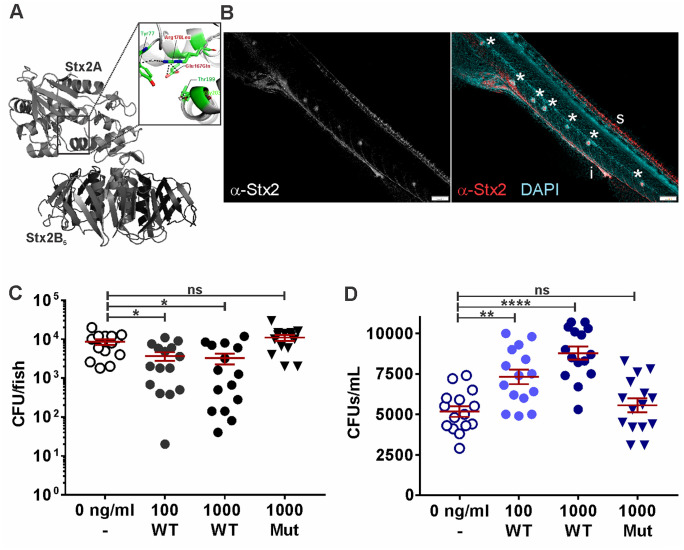
Shiga toxin 2 is sufficient to cause partial loss of the intestinal microbiota. **(A)** Stx2AB_5_ structure (A subunit in purple, B subunits in green) and position of mutated residues (box) in enzymatically inactive mutant Stx2A E167Q, R170L. Structure: PDB 1R4P [25]. Non-EHEC CFUs recovered from **(B)** 7 dpf larva immersed in 1000 ng/ml Stx2 for 24 hrs; immunofluorescence staining w a-Stx2 and DAPI shows Stx2 is enriched in the intestinal tract **(i)**, spinal cord **(s)**, and lateral line neuromasts (*). Scale bar 100 µm. **(C)** larval tissue homogenates and **(D)** shed into the fish media at 7 dpf following 24 hrs of immersion in 100 – 1000 ng/ml wild type purified Stx2AB or 1000 ng/ml inactive Stx2AB mutant (Mut) compared to untreated fish (0 ng/ml). Data points represent individual larvae or media samples (N = 15), and means ± sem (red); Statistics: ANOVA and Dunnett’s multiple comparison test, ****p ≤ 0.0001, **p ≤ 0.01, *p < 0.05, ns (not significant) p ≥ 0.05.

Next, we studied if Stx2 alone would cause increased shedding of endogenous microbiota constituents from the intestine. We purified and reconstituted recombinant wild type Stx2 complex according to a published protocol [24], as well as the previously characterized enzymatically inactive complex containing Stx2A E189Q, R192L, equivalent to Stx2A E167Q, R170L in Fig 3A [25], see Methods section), and exposed fish to Stx2 by immersion in E3 medium containing doses of 100–1000 ng/ml toxin. The LD_50_ of Stx2 in zebrafish has previously been determined to be 33 µg/ml [26], and the range of concentrations used here had no effect on growth, development, morbidity, or mortality of the fish. Following larval Stx2 exposure by immersion for 24 hours, Stx2 was enriched in the zebrafish intestinal tract, spinal cord, and lateral line neuromasts (Fig 3B). Exposure of fish to 100–1000 ng/ml wild type toxin led to an approximately 3-fold drop in colonization with endogenous microbiota, while bacterial colonization remained unchanged following exposure to enzymatically inactive Stx2 (Fig 3C). This drop in intestinal burden was reflected by increased amounts of shed bacteria recovered from the fish media following exposure to wild type, but not enzymatically inactive toxin (Fig 3D). These results indicate that exposure of zebrafish to Stx2 is sufficient to increase shedding of the endogenous host microbiota, and that this effect depends on Stx2A enzymatic activity but does not require phage-mediated lysis.

### Stx2 alters the composition but not the diversity of the zebrafish gut microbiome

**Fig 4:**
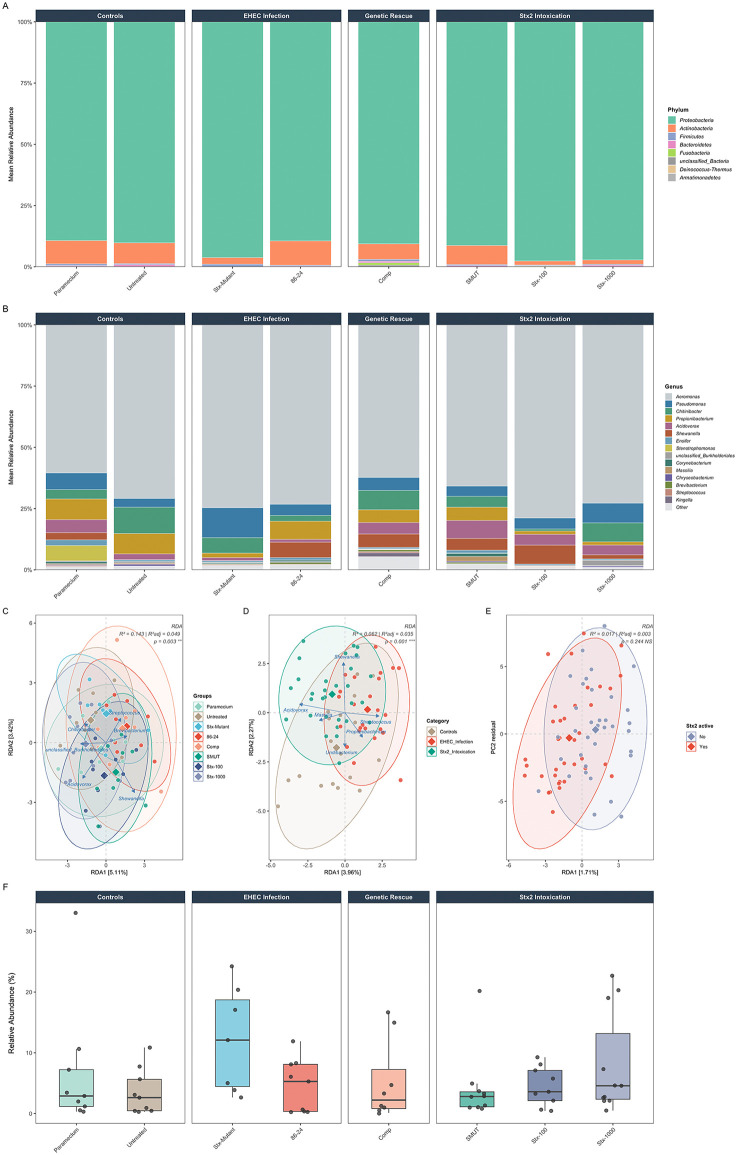
STEC infection and Stx2 treatment changes the zebrafish intestinal microbiome structure. **(A)** Phylum-level relative abundance of the microbiome across experimental groups. Stacked bar plots show mean Phylum-level relative abundance per group, organized by experimental category (Controls, EHEC Infection, Stx2 Intoxication). **(B)** Genus-level relative abundance of the gut microbiome across experimental groups. Stacked bar plots show mean relative abundance of the 15 most abundant genera per group. **(C-E)** Canonical RDA on CLR-transformed genus abundances. **(C)** Constrained by group identity (R² = 0.143, p = 0.002); blue arrows = top 6 genus loadings. **(D)** Constrained by experimental category (R² = 0.062, p = 0.002); Stx2 Intoxication separates along RDA1 driven by *Acidovorax* and *Massilia*. **(E)** Constrained by Stx2 activity status (R² = 0.017, p = 0.258 NS); active and inactive groups completely overlap. Diamonds = group centroids; 95% confidence ellipses shown. **(F)** *Pseudomonas* relative abundance across experimental groups. Δ*stx2*: 12.19%, 86-24: 4.57%, Comp: 5.28%. Directionally consistent with Stx2-dependent displacement. Boxes show the median (center line), interquartile range (IQR; box edges), and 1.5 × IQR (whiskers). Individual data points are shown; outliers beyond 1.5 × IQR are plotted individually.

While the above-described experiments clearly indicate depletion of the endogenous intestinal microbiota, our ability to monitor changes in the microbial structure in these experiments is limited because only a limited number of species grow on EHEC CHROMagar. Thus, we carried out a more comprehensive, culture-independent analysis of the intestinal microbiome structure and changes caused by EHEC infection or Stx2 treatment alone, using 16S rRNA sequencing. We sequenced tissues from fish fed paramecia alone (uninfected), or infected with 86-24 EHEC wild type, *∆stx*2 mutant or complemented strains. We also sequenced the microbiome of fish treated with 1000 ng/ml enzymatically inactive Stx2, and compared the composition to fish treated with 100–1000 ng/ml active toxin (Fig 4).

After quality filtering, 72 samples were retained: Paramecium (n = 9), Untreated (n = 9), Δstx2 (n = 7), 86-24 (n = 9), Comp (n = 8), SMUT (n = 10), Stx-100 (n = 9), Stx-1000 (n = 11). *Proteobacteria* dominated all groups, at the phylum level (89-98%; Fig 4A). Infection with wild-type EHEC and Δstx2::stx2-complemented strains induced modest *Firmicutes* expansion compared to control and Δ*stx2*, likely reflecting an inflammatory response and colonization. In contrast, Stx2 toxin alone (Stx-100, Stx-1000) specifically reduced *Actinobacteria* (1.6–1.8% vs. 8–10% in controls) without the *Firmicutes* expansion seen during EHEC infection. This dissociation suggests that Stx2-mediated microbiome changes are not simply a consequence of bacterial colonization.

At the genus level (Fig 4B), *Aeromonas* dominated all conditions (60–79%) and showed no consistent Stx2-dependent pattern. *Chitinibacter* (*Burkholderiales*) was notably lower in 86-24 (2.34%) and Stx-100 (0.84%) compared to Untreated fish (10.8%). Neither EHEC infection nor Stx2 exposure, whether as live bacteria or purified toxin, significantly alters within-sample diversity (Kruskal-Wallis, p > 0.6; Fig HA in S1 Appendix). Across all analyses, *Pseudomonas* abundance follows a consistent directional pattern aligned with Stx2 activity (Figs 4F and HE in S1 Appendix): highest when Stx2 is absent (Δ*stx2*: 12.19%), intermediate in controls (3.57–6.81%), and lowest when Stx2 is present whether through infection (86-24: 4.57%, Comp: 5.28%) or purified toxin (Stx-1000: 8.05% vs. SMUT: 4.19%). Pairwise Wilcoxon tests were non-significant after Benjamini-Hochberg (BH) correction (all p_adj > 0.05), with the Δ*stx2* vs. 86-24 comparison showing a strong trend (raw p = 0.090). *Aeromonas*, the dominant genus (60–79%), maintained stable abundance across all conditions including high-dose Stx2. *Shewanella* showed no consistent displacement. Among the five most abundant genera, only *Pseudomonas* and *Acidovorax* showed Stx2-dependent reduction. *Chitinibacter* (*Burkholderiales*) varied across conditions (10.8% Untreated vs. 2.34% 86-24) but less consistently than *Pseudomonas*. The microbiome is restructured at the genus level without losing overall species richness or evenness, consistent with partial displacement of select taxa rather than global community collapse.

Community structure differed significantly across all 8 groups (PERMANOVA R² = 0.143, p = 0.002; Fig HB in S1 Appendix) and by experimental category (R² = 0.062, p = 0.001; Fig HC in S1 Appendix). Group identity accounts for a significant fraction of variation in community structure (R² = 0.143, p = 0.003), while experimental category was also significant (R² = 0.062, p = 0.002) (Fig 4C-4E). However, Stx2 activity status alone, comparing Stx2-active groups (86-24, Δstx2::stx2, Stx-100, Stx-1000) against Stx2-inactive groups (Paramecium, Untreated, Stx-Mutant, SMUT), did not significantly constrain community structure (R² = 0.017, p = 0.258 NS).

Together, these results show that Stx2 does not cause global community restructuring. Rather, toxin-mediated effects are taxon-specific rather than community-wide.

### *Shiga toxin 2 facilitates displacement of endogenous* Pseudomonas ssp. *from the intestine*

The 16S rRNA sequencing results suggested taxon-specific changes in abundance following infection or Stx2 exposure. To validate the microbiome sequencing results, we conducted plating-based experiments. We chose *Pseudomonas* for these follow-up experiments, since *Pseudomonas* abundance follows a consistent pattern aligned with Stx2 activity, and changes in *Pseudomonas* abundance can easily be tracked by plating tissue homogenates in selective medium (Cetrimide agar). Our attempts to isolate gut isolates from other species with large changes and/or find a selective medium for enumerating them, were less successful.

**Fig 5:**
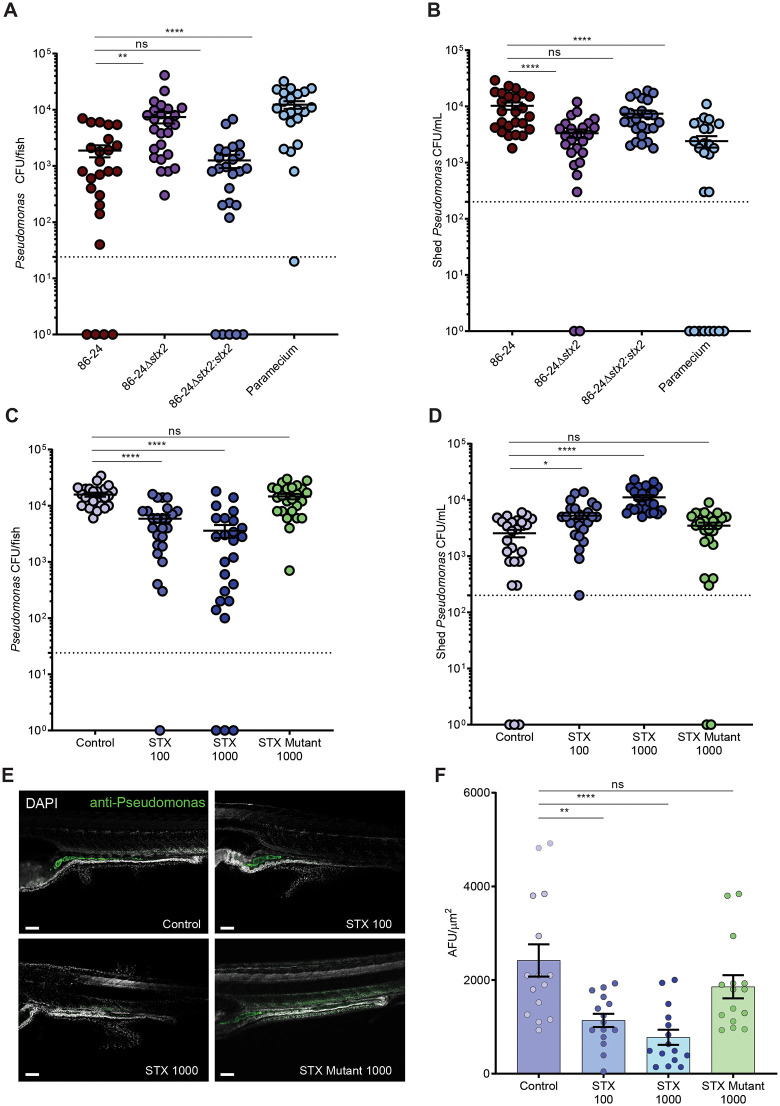
Shiga toxin 2 facilitates displacement of endogenous *Pseudomonas ssp.* from the intestine. *Pseudomonas* CFUs recovered from larval tissue homogenates **(A, C)** or shed into the fish media **(B, D)** at 7 dpf following food-borne infection (A, B) or Stx2 toxin immersion (C, D) of larvae for 24 hrs. Controls were larvae fed paramecia vehicle without EHEC (para). Data are individual fish or media samples (N = 25), and means ± sem (red); **(E)** Immunofluorescence staining (α-*Pseudomonas* antibody green, DAPI blue) of 7 dpf larvae immersed in Stx2 for 24 hrs. Scale bar 100 µm. **(F)** Quantification of *Pseudomonas* (green) fluorescence intensity in the intestine of imaged fish. Data are means ± sem (N = 15 fish/condition). Statistics: ANOVA and Dunnett’s multiple comparison test, ****p ≤ 0.0001, **p ≤ 0.01, *p < 0.05, ns (not significant) p ≥ 0.05; Samples with no detectable bacterial burden were plotted as 1 for visualization and statistical analysis. Dashed line = limit of detection.

Infection of fish with EHEC wild type and complemented strains significantly decreased intestinal colonization with *Pseudomonas ssp*., while *Pseudomonas* colonization in fish infected with EHEC *Δstx*2 was similar to uninfected fish (Fig 5A). In line with these results, exposure to recombinant wild type Stx2 caused a significant decrease in *Pseudomonas* colonization, while levels remained unchanged in fish exposed to Stx2 mutant (Fig 5C). Shedding of *Pseudomonas* into the fish medium was low in uninfected (paramecia vehicle only) fish and fish infected with EHEC *Δstx*2, but significantly increased in fish infected with EHEC wild type or *Δstx*2*:stx*2 complemented strains (Fig 5B). Similarly, treatment of fish with 100–1000 ng/ml purified Stx2 significantly enhanced shedding of *Pseudomonas*, compared to untreated fish and fish exposed to the enzymatically inactive toxin (Fig 5D). Additionally, we confirmed these results by immunostaining untreated fish and fish exposed to purified Stx2 toxin with *Pseudomonas*-specific antibody. In untreated fish, we observed antibody staining for *Pseudomonas* throughout the zebrafish intestinal lumen (Fig 5E). Quantitative analysis of the staining showed that colonization with *Pseudomonas* significantly decreased in fish treated with wild type Stx2, but not in those exposed to inactive Stx2 (Fig 5D and 5E). Together, these data corroborate the results from 16S rRNA sequencing and demonstrate that Stx2 is necessary and sufficient to decrease colonization and increase shedding of endogenous *Pseudomonas* species from the zebrafish gut.

### Shiga toxin 2 accelerates intestinal transit

**Fig 6:**
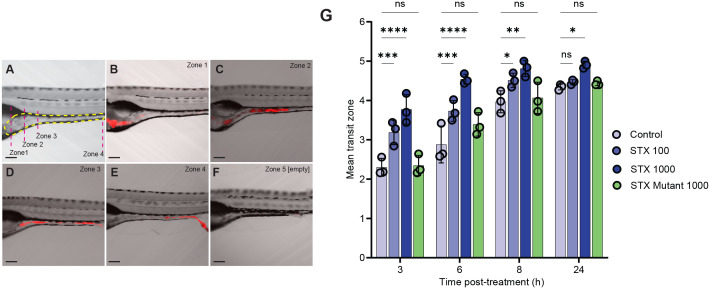
Shiga toxin 2 accelerates intestinal transit. **(A)** Location and boundaries of zones 1-4 relative to the intestine used for scoring intestinal transit, and examples of 7 dpf larvae fed fluorescent tracer with food transiting zones **(B)** 1, **(C)** 2, **(D)** 3, **(E)** 4, and **(F)** empty gut. Scale bars 100 µm. **(G)** Gut transit assay. Fish were fed fluorescent tracer food and immersed in vehicle control (CTL), 100-1000 ng/mL WT Stx2, or 1000 ng/ml Stx2 mutant, and tracer location was scored at 3, 6, 8, and 24 hpi. Data are means ± sem from N = 3 independent experiments (N = 50 fish/group); Statistics: two-way ANOVA and Dunnet’s test. ****p ≤ 0.0001, ***p ≤ 0.001, **p ≤ 0.01, *p < 0.05, ns (not significant) p ≥ 0.05.

We observed that Stx2 displaces endogenous *Pseudomonas sp*. from the zebrafish gut by increasing bacterial shedding, but has no antimicrobial activity against *Pseudomonas*. To uncover the mechanism behind this displacement, we tested whether Stx2 accelerates gut transit, leading to faster passage of luminal contents. Fish were fed food containing a fluorescent tracer, and gut transit was measured by imaging and scoring as previously described [27,28], Fig 6A-6F). We found that gut transit was significantly sped up in fish treated with 100–1000 ng/ml Stx2. In contrast, fish treated with 1000 ng/ml enzymatically inactive Stx2 exhibited transit rates comparable to untreated controls (Figs 6G and 6E in S1 Appendix).

Beyond accelerating gut transit, Stx2 is known to impact cell physiology in several other ways. It can promote cell death [29,30] and trigger proinflammatory responses [31,32], which might also contribute to the observed increase in bacterial shedding. To test whether accelerated gut transit alone could explain the shifts in microbiota colonization and shedding seen with Stx2, we treated fish with 0.2-1 mM caffeine, a known prokinetic agent that enhances gut motility [33,34]. Both caffeine doses significantly increased gut transit, with effects detectable as early as 2 hours after exposure and lasting up to 24 hours – mirroring the impact of Stx2 treatment (Fig F in S1 Appendix). Both caffeine and Stx2 treatment caused a similar decrease in *Pseudomonas* gut colonization (Fig GA in S1 Appendix), and a parallel increase in shedding of viable *Pseudomonas* into the E3 medium (Fig GB in S1 Appendix).

Together, these findings suggest that Stx2 boosts bacterial shedding by accelerating gut transit, and that this toxin-driven change in host physiology is sufficient to cause the partial displacement of endogenous gut microbiota.

## Discussion

It has long been clear that Stx is an important virulence factor with multifaceted contributions to host pathology. Its cytotoxic activity promotes damage to the epithelium, vasculature, and central nervous system, contributing to host morbidity and mortality. Although Stx is evolutionarily well conserved, how it benefits bacterial fitness has remained unclear. Some studies suggest that Stx carriage confers an advantage during environmental persistence against amoeboid predators [19,21,35]. Here, we show that Stx2 facilitates intestinal colonization in the zebrafish model (Fig 1). We employed a food-borne EHEC infection model that uses internalization of EHEC by the protozoan *Paramecium caudatum*, followed by feeding larval zebrafish with EHEC-loaded paramecia for 2 hours. This approach enhances bacterial intestinal burden compared to infection via direct immersion of larvae in EHEC suspensions [11]. Our findings align well with previous reports demonstrating Stx-dependent differences in colonization in a conventional mouse model [8]. The observation that co-colonization with a wild type strain rescues the colonization defect of a Stx-deficient strain, both in mice [8] and zebrafish, strongly supports a fitness advantage mediated by the secreted toxin.

In contrast to prior studies reporting that Stx prophage carriage affects bacterial survival within grazing protozoans such as *Tetrahymena* [20,21], we found no effect of Stx on bacterial persistence within *Paramecium* (Fig AA in S1 Appendix). This discrepancy could stem from differences in protozoan host species or the shorter time frame studied here (up to 6 hours versus several days in other studies).

Our results support a role for Stx2 in enhancing EHEC colonization fitness in the zebrafish gut, consistent with mammalian models. Deletion of *stx2* in both EDL933 and 86-24 strains caused significantly reduced bacterial burden, a phenotype fully rescued by chromosomal complementation or co-colonization with wild type strains, indicating that secreted Stx2 can function in trans. These effects are not due to differences in bacterial dose or growth, as uptake by *Paramecium* and *in vitro* growth rates remained consistent across strains.

Plating-based approaches provide only limited insight into the resident microbiome: EHEC CHROMagar suppresses Gram-positive growth, and our aerobic incubation excludes less aerotolerant species. To better elucidate how Stx facilitates colonization, we combined culture-dependent plating assays (Fig 2) with culture-independent 16S rRNA sequencing (Fig 4). Our decision to focus on plating for *Pseudomonas* spp. was based on its ranking among the top five most abundant genera across samples and the availability of Cetrimide agar as a selective medium. We successfully isolated multiple *Pseudomonas* strains from the zebrafish gut for further assays, including phage lysis and toxin sensitivity tests. The five *Pseudomonas* strains used here represent three distinct OTUs covering over 99.9% of *Pseudomonas* reads in our samples. However,further characterization of other abundant genera (*Aeromonas*, *Propionibacterium*, *Acidovorax*, and *Chitinibacter*) was limited by the absence of suitable selective media. Both plating and sequencing approaches converge on a role for Stx in reshaping the gut microbial community in ways that benefit EHEC. This is consistent with previous work showing Stx is dispensable for colonization in germ-free mice [9].

In zebrafish, EHEC infection primarily led to an expansion of the phylum Firmicutes (Fig 4A), whereas exposure to Stx2 alone caused an increase in Proteobacteria and a decrease in Actinobacteria (Fig 4A). This dissociation suggests that Stx2-mediated microbiome changes are not simply a consequence of bacterial colonization and competition. At the genus level, Aeromonas dominated and maintained stable abundance across all conditions. Among the five most abundant genera, only *Pseudomonas* and *Acidovorax* showed Stx2-dependent depletion. Notably, no significant changes in alpha diversity were observed, but community structure differed significantly across all experimental groups (Figs 4 and H in S1 Appendix). Differences between infection and Stx intoxication groups might be due to the inflammation caused by the infection, or additional EHEC virulence factors that together dominate the effect on the microbiome. However, the consistent decrease in *Pseudomonas* between infection and Stx2 treatment highlights its robustness as a target taxon for further plating-based studies. Furthermore, the different *Pseudomonas* strains we isolated from the zebrafish gut and used for follow-up studies represent three distinct OTUs, which together account for more than 99.9% of all *Pseudomonas* reads in our samples. We can therefore conclude that results obtained with these strains are representative of the majority of *Pseudomonas* strains present in the microbiome.

EHEC infections in humans and cattle similarly disrupt native gut microbiota, causing dysbiosis [36–38]. Stx2 has been implicated as a colonization-enhancing virulence factor in cattle [39,40]. STEC-associated dysbiosis in patients features increases in Proteobacteria and Actinobacteria, and a decrease in Bacteroidetes [36]. The cattle microbiome differs markedly, with genera such as *Ruminococcus* and *Bacteroides* enriched in EHEC super-shedders, while *Prevotella* is more abundant in non-shedders (33). These host-specific microbial signatures influence colonization dynamics. The zebrafish core microbiome differs substantially in complexity and composition from human microbiomes [41], limiting the direct translatability of phylum-level changes. For example, EHEC infection decreases Firmicutes in humans [36] but increases them in zebrafish (Fig 4). Nonetheless, both models demonstrate that infection induces significant microbiome remodeling, consistent with studies showing that a robust microbiome protects against EHEC colonization [42,43].

To separate direct toxin effects on the microbiome and host physiology from other infection-associated cues that may potentiate toxin activity (for example the release of lipopolysaccharide, [44], we administered purified Stx2 to zebrafish. We administered doses of 100–1000 ng/ml, which we determined to be equivalent to the concentrations released into fish during EHEC infection. Immunostaining for Stx showed enrichment of the toxin in the intestinal tract, spinal cord and lateral line neuromasts, structures rich in sensory neurons. This pattern is consistent with published reports in mammalian hosts showing neurotoxicity following toxin release in the intestine [3]. A prior study determined an LD_50_ of 33 µg/ml for Stx2 in larval zebrafish by immersion, with intoxication leading to edema [26]. Our sublethal doses induced no morbidity or mortality but revealed Stx2’s impact on intestinal motility and transit as a likely mechanism mediating bacterial competition (Fig 6). Gut motility is a key driver shaping the gut ecosystem and modulating microbe-microbe interactions [45]. Some enteric bacteria promote increased gastric motility via tryptophan metabolites [46]. In *Vibrio cholerae*, the type 6 secretion system modulates intestinal mechanics to compete with resident gut microbes [47,48]. However, increased intestinal activity has not previously been proposed as a mode of Stx action. Enteric infections can boost motility through serotonin signaling changes [49,50]. Yet so far, we have found no evidence that Stx2 elevates serotonin in zebrafish and its mechanism of action is subject to ongoing studies. Stx2 has been reported to activate reactive glial cells in the hippocampus [3], and it is plausible that enteric glial cells are similarly affected. Enteric glial cells have been shown to regulate gut motility, and their dysfunction results in altered gut transit times and impacts microbial community dynamics [51,52]. The fact that Stx is enriched in the intestinal tract also gives rise to the possibility that it affects viability and shedding of intestinal cells, which could be an additional mechanism contributing to the loss of microbiota associated with those cells.

Despite these complex physiological effects, our caffeine experiments demonstrate that accelerated gut transit alone is sufficient to drive *Pseudomonas* displacement via expulsion (Figs G and H in S1 Appendix). This aligns with reports that drugs altering gut motility cause dysbiosis in zebrafish microbiota [53] and that caffeine affects microbiota composition in murine models [54].

In conclusion, our study uncovers a novel link between Stx2 and host intestinal transit, promoting EHEC competition with the resident gut microbiota and enhancing colonization fitness. While we highlight a plausible mechanism by which Stx2 benefits bacterial fitness, its mechanism of action in the gut remains to be elucidated. Future work dissecting these pathways could reveal novel therapeutic targets. Moreover, exploring the potential of counteracting Stx2's impact on the intestinal microbiota through probiotic or prebiotic interventions may provide practical strategies for maintaining gut health during EHEC infection.

## Materials and methods

### Ethics statement

Zebrafish care, breeding, and experiments described here are in accordance with the Guide for the Care and Use of Laboratory Animals have been approved by the Institutional Animal Welfare Committee of the University of Texas Health Science Center, Houston, and protocol number AWC-22–0088.

### Bacterial strains, plasmids, and growth conditions

A list of strains and plasmids can be found in Table 1 and Table A in S1 Appendix. The EHEC wild type strains used were serotype O157:H7 EDL933 and 86-24. Deletion strains were constructed by recombineering using the gene doctoring system as previously described [55]. Deletions were complemented by transposon insertion of *stx*2*ab* at the attB site, as previously described [56], and cytotoxicity of cell-free supernatants from bacterial cultures was tested to validate the presence or absence of toxin production. Please note that the Stx2A enzymatically inactive point mutant annotated as Stx2A E167Q, R170L in structure PDB 1R4P [25] is equivalent to our point mutant Stx2A E189Q, R192L, and nomenclature is different because the PDB structure lacks the first 22 residues at the N-terminus of Stx2A due to post-translational processing/cleavage, and residue 23 is labeled as 1 in the structure.

### Bacterial growth assays

Bacterial cultures were inoculated from single colonies into LB and grown at 37°C shaking at 200 rpm for 16 hours. Cultures were diluted 1:100 into fresh LB in 96-well plates, and cultures grown at 37°C shaking at 120 rpm inside a FluoStarOmega spectrophotometer (BMG Lab), and OD600 was measured every hour for 24 hours. Blank media (LB only) was used for baseline correction and experiments were run in triplicate. To evaluate potential effects of recombinant, purified Stx2 on bacterial growth, cultures were grown as above, and diluted 1:100 into LB containing 1 µg/ml Stx2, or LB containing an equivalent volume of E3 media (since toxin was dissolved in E3 media) as a control. To test if fish shed any soluble compounds into E3 media that could affect bacterial growth, shedding media was collected after infections, and sterilized by passing through a 0.2 µm pore size syringe filter. As a control, E3 media was used. Bacteria were grown as above, and cultures were diluted 1:100 into a 1:1 mixture of LB and either sterilized shedding media, or fresh E3 media, and OD600 was measured as described above.

### Phage plaque assay

Phage plaque assays were adapted from a previously published protocol [57], with some modifications as follows: Overnight cultures of strains EDL933 and 86-24 were diluted to an OD600 of 0.05, and incubated for 1.5-2 hours until the OD600 reached 0.4-0.5. LB alone was used as a control. Each culture was divided, with one part treated with 1 µg/ml mitomycin C and the other left untreated as a control. Cultures were incubated at 37°C for 6–7 hours, and the OD600 was monitored hourly. When the OD600 of the cultures dropped to 0.2 or lower, phage particles were isolated by centrifuging the cultures at 10,000 rpm for 10 minutes at 4°C. The supernatant was filtered twice using a 0.22 µm PES syringe filter, and serial dilutions of the phage lysates were prepared in SM buffer (200 mM NaCl, 10 mM MgSO₄, 50 mM Tris pH 7.5, 0.01-0.02% gelatin w/v). Plaque assays were performed using the double agar overlay method using 1.5% agar as the bottom layer. As indicator cells, freshly grown MG1655 or *Pseudomonas* isolates zfem002–005, and overnight-grown *Pseudomonas* isolate zfem001 were used. For the top layer, 335 µl of indicator bacteria, 1 ml of 100 mM CaCl₂ (final concentration 10 mM), 1 ml of 100 mM MgCl₂ (final concentration 10 mM), and 7.5 µl of 2 mg/ml mitomycin C (final concentration 1.5µg/ml), were mixed thoroughly with 8 ml of soft agar (0.7% agar, 50°C) and poured onto the bottom layer. Phage lysates and serial dilutions thereof (5 µl each) were spotted onto the plates, along with LB containing mitomycin C as a negative control. The plates were incubated at 37°C overnight before imaging.

### Cell-based cytotoxicity assay

Cytotoxicity of *E. coli* strains toward Vero cells (see Table 1) was assessed following the approach described in [58], using Vero cells (ATCC CCL-81) cultured in DMEM supplemented with 10% FBS. Cell-free supernatants were prepared from bacterial cultures as described [58], and were applied to confluent monolayers and incubated for 24 hours at 37°C with 5% CO_2_. Cell viability was quantified using LDH release assays (Pierce), as detailed in the manufacturer’s protocol, and three biological replicates were run. To facilitate comparison across strains, cytotoxicity was summarized using the following scoring system: - (no cytotoxicity, 0–9% lysis); + (mild cytotoxicity, 10–29% lysis); ++ (moderate, 30–59% lysis); +++ (severe cytotoxicity, > 60% lysis).

### Zebrafish maintenance

Zebrafish care, breeding, and experiments described here are in accordance with the Guide for the Care and Use of Laboratory Animals have been approved by the Institutional Animal Welfare Committee of the University of Texas Health Science Center, Houston, and protocol number AWC-22–0088. The zebrafish used in this study were wild type AB. Adult fish were kept in a recirculating tank system at the University of Texas Health Science Center at Houston Laboratory Animal Medicine and Care on a 14:10 h light: dark cycle at pH 7.5 and 28°C. Eggs were obtained from natural spawning of adult fish. Fertilized embryos were bleached for 30 sec. in 0.05% sodium hypochlorite solution and kept at 30°C on a 14:10 h light:dark cycle at pH 7.4. Embryos were raised in E3 media (10 mM HEPES, 5 mM NaCl, 0.17 mM KCl, 0.4 mM CaCl_2_, 0.67 mM MgSO_4_, pH 7.4). Starting at 5 days post fertilization (dpf), larvae were fed GEMMA Micro 75 (Skretting) once per day at the beginning of the light cycle. Larvae were moved to fresh E3 daily.

### Food-borne colonization of larval zebrafish with EHEC, bacterial burden and shedding

A detailed protocol for food-borne infection of larval zebrafish has been published previously [13]. Briefly, larvae at 7 dpf were infected with approximately 7 x 10^5^ CFUs of EHEC per fish, using *Paramecium caudatum* as a vehicle. *P. caudatum* are incubated in a suspension of EHEC for 2 hours to achieve bacterial internalization. *Paramecia* are then washed and fed to larval fish for 2 hours, followed by transfer of larvae to fresh E3 and euthanized 24 hours post-infection. Uninfected fish were fed paramecia only without EHEC. For co-colonization experiments, the same bacterial dose was used, but consisting of a 1:1 mixture of both strains (∆*stx*2:mcherry^tetR^ and unmarked WT). 24 hours post colonization, larvae were euthanized by immersion in 1.6 mg/ml buffered tricaine pH 7.5, washed in PBS, and individual larvae were resuspended in 120 µl of sterile 1 mg/ml pronase solution. Tissue samples were further disrupted by needle homogenization using 31-gauge needles. Serial dilutions of the homogenates were plated on CHROMagar O157 and plates were incubated at 37°C for 20 hours, followed by enumeration of EHEC CFUs (visible as mauve colonies), and non-EHEC CFUs (blue and white colonies). For co-colonization assays, diluted homogenates were plated on LB agar containing 10 µg/ml tetracycline, and total CFUs as well as mCherry positive CFUs were determined. Note that some of the natural zebrafish gut microbiota is tetracycline resistant. For enumeration of bacteria shed into the medium during infection, media samples were taken at 24 hpi, serially diluted, and plated as described above. To enumerate *Pseudomonas* CFUs, samples were plated on Cetrimide agar. The limit of detection for this assay is 24 CFU/fish, and 200 CFU/ml for shedding media, respectively. Samples containing CFU counts below the detection limit were assigned a CFU of 1 for data visualization and statistical analysis. Data analysis was carried out using GraphPad Prism software, version 9.

### Paramecia maintenance and burden of *E. coli* inside of paramecia

*Paramecia* were propagated one day prior to the infection experiment and every 2 weeks to maintain live cultures, as previously described [13]. On the day of the experiment, paramecia were co-cultured with *E. coli* to promote bacterial internalization, and the amount of *E. coli* inside of the paramecia at different time points was assessed by lysing the paramecia with 1% Triton X-100 followed by colony forming unit (CFU) dilutions and plating. In addition, the number of paramecia at each time point was determined using an automated cell counter (Life Technologies Countess II), and used to determine CFUs/paramecia at each time point.

### Immunofluorescence staining and imaging of zebrafish

The larvae were euthanized and fixed overnight in 4% formaldehyde at 4°C. After fixation, they were washed twice with 1X PBS, permeabilized in acetone for 15 minutes at -20°C, and then blocked overnight in PBDT blocking solution (PBS, 1% BSA, 1% DMSO, 0.5% Triton-X100). Subsequently, the larvae were incubated overnight at 4°C with anti-*Pseudomonas* antibody (Thermo Scientific, PA173116) or anti-STX antibody (GeneTex, GTX43055) at a 1:50 dilution. The following day, samples were washed and incubated with goat anti-rabbit IgG Alexa Fluor 488 (Thermo Fisher Scientific, A27034) or with anti-mouse secondary antibody CF594 Red (Sigma, SAB4600402) at a 1:250 dilution and 1 µg/mL 4′,6-diamidino-2-phenylindole (DAPI), overnight at 4°C. After incubation, the samples were washed three times for 30 minutes with a wash buffer (1X PBS, 0.1% Tween-20, 0.1% Triton X-100). Larvae were embedded in low melt agarose and imaged using an Olympus Fluoview FV3000 confocal microscope at 60X magnification, and images were processed with cellSENS version 2.3 software using five iterations of deconvolution.

### Protein expression and purification

Expression and purification of recombinant His-tagged Stx2AB was carried out following a previously published protocol [24]. The His-tag is located at the C-terminus of Stx2B. Briefly, *E. coli* BL21(DE3) transformed with pBADmycHis-stx2ab or pBADmycHis-stx2ab E189Q R192L was grown in LB broth containing 100 µg/ml ampicillin overnight at 37°C. Buffers were prepared as follows: binding/wash buffer comprised 25 mM Tris-HCl (pH 7.5), 150 mM NaCl, and 5 mM imidazole; elution buffers included E100 (100 mM imidazole), E200 (200 mM imidazole), and E500 (500 mM imidazole), all in 25 mM Tris-HCl (pH 7.5). 1 mL of the overnight culture was inoculated into 100 mL of LB medium containing ampicillin and incubated at 37°C with shaking for 2 hours, reaching an OD600 of 0.2–0.5. Protein expression was induced with 0.25% sterile L-arabinose and further incubated for 4 hours. Cells were pelleted by centrifugation and stored at -20°C overnight. For lysis, the cell pellet was thawed on ice, resuspended in a lysis buffer consisting of wash buffer, lysozyme (2 mg/mL), Roche complete protease inhibitor cocktail, and 5 mM MgCl2, and incubated for 30 minutes with rotation at 4°C. Cells were then sonicated for a total of 24 minutes and centrifuged at 17,000 rcf at 4°C to clarify the lysate. The pH of the lysate was verified to be approximately 7–7.5. The clarified lysate was loaded onto a gravity column pre-equilibrated with binding buffer, followed by washing with wash buffer and elution using E100, E200, and E500 buffers. Eluted fractions containing Stx were combined and dialyzed overnight in PBS (2 L) at 4°C with stirring using SnakeSkin Dialysis Tubing (3.5 kDa MWCO).

### Exposure of larval zebrafish to recombinant Stx2 or caffeine

Larvae at 7 dpf were placed in E3 medium containing 100 ng/ml or 1000 ng/ml purified, recombinant Stx2 or Stx2 E189Q R192L mutant (diluted into E3 from 1 mg/ml protein stock in PBS) or in E3 alone (untreated), and maintained at 30°C for up to 24 hours, prior to euthanizing. In prior experiments, we determined that Stx2 exposure for 24 hours at the above concentrations did not cause any mortality. Mortality was accessed by visual observation, and the absence of operculum movement. To evaluate the effects of caffeine on zebrafish gut motility and bacterial colonization/shedding, larvae at 7 dpf were placed in E3 medium containing 200 µM or 1 mM caffeine, and gut transit assessed as published [28].

### Microbiome DNA extraction and 16S rRNA gene sequencing

The larvae were euthanized and whole, individual zebrafish were used for DNA extraction using the Qiagen PowerSoil Kit following the manufacturer’s protocol (Qiagen, 12888). 16S rRNA library preparation, sequencing, and data processing was performed as described previously [59]. Briefly, we amplified the V1-V3 region of the 16S rRNA gene utilizing unique dual barcodes for each sample, and sequenced the libraries using an Illumina MiSeq and a v3 2x300 sequencing kit (Illumina, MS-102–3003).

### Bioinformatic processing

De-multiplexed 16S rRNA gene sequencing reads were filtered, trimmed, denoised, and merged using DADA2 (v1.36.0) [60] in RStudio (v4.5.0). The parameters used to trim and filter the reads were TruncLen (295,255) and MaxEE (2,5) from DADA2’s filterAndTrim() function. The RefSeq v2 database [61] (https://benjjneb.github.io/dada2/training.html) and the AssignTaxonomy() function were used for the taxonomic assignment, which was later integrated with the OTU table and the metadata file and imported into a Phyloseq object using Phyloseq (v1.52.0) [62].

### Quality control

Samples with fewer than 5,000 reads after quality filtering were excluded (13 of 85 samples removed, leaving n = 72). This threshold was established based on read-depth analysis showing that low-depth samples exhibited artifactual diversity inflation (Spearman ρ = −0.292, p = 0.007 for depth vs. Shannon in unfiltered data) and their inclusion obscured genuine group structure in PERMANOVA (p = 0.238 unfiltered vs. p = 0.041 filtered;). Following quality filtering, the depth-diversity correlation was reduced to non-significance (ρ = −0.134, p = 0.261).

#### Bioinformatics statistical analysis.

All bioinformatics statistical analyses were performed in R (v4.5). Four alpha diversity metrics were calculated: Observed ASVs, Chao1, Shannon, and Simpson. Differences were assessed by Kruskal-Wallis tests at both the 8-group and 4-category levels, with pairwise Wilcoxon rank-sum post-hoc comparisons. Community dissimilarity was assessed using Aitchison distance on centered log-ratio (CLR) transformed abundances (pseudocount = 0.5). CLR transformation was chosen over standard PCoA/Bray-Curtis because the untransformed compositional data exhibited a classical horseshoe artefact in ordination space. PCA on CLR-transformed data was used for ordination. PERMANOVA (adonis2, 999 permutations) tested community structure at the group level (8 groups) and category level (4 categories: Controls, EHEC Infection, Genetic Rescue, Stx2 Intoxication). Redundancy analysis (RDA) partitioned variance attributable to group identity, biological category, and binary Stx2 activity status. Constrained correspondence analysis (CCA) using chi-square distances on relative abundances provided independent methodological confirmation. Beta dispersion (betadisper) confirmed homogeneous within-group variance, validating PERMANOVA interpretation.

Two complementary differential abundance approaches were used: (1) Kruskal-Wallis with Dunn post-hoc (BH-adjusted) on relative abundance data, and (2) Welch t-tests on CLR-transformed abundances to correct for compositionality, both with Paramecium vehicle control as reference. Targeted *Pseudomonas* analysis used pairwise Wilcoxon rank-sum tests with BH correction for four pre-specified comparisons (Δ*stx2* vs. 86-24, Δ*stx2* vs. Δ*stx2:stx* complement (Comp), Untreated vs. Stx-1000, Untreated vs. 86-24).

### Preparation of fluorescent food and transit assay

Fluorescent tracer food was prepared as described in [27], with minor modifications. Ratios of food and tracer were maintained as per the published protocol but as food, we used Gemma micro 75 ZF (Skretting) and spiked it with fluorescent red, amine-modified polystyrene beads (1 µm mean particle size, Sigma L2778). From 5 dpf, fish were fed unlabeled Gemma twice daily. At 7 dpf, batches of 100 fish per group were fed 2mg tracer food for 2 hours, followed by sorting of fish into batches of n = 50 fish/group which had a tracer filled bulb. Fish were left in E3, or E3 containing Stx as indicated, and imaged and scored at 3, 6, 8, and 24 hours post exposure. Transit zones were scored visually, following the scoring scheme described in [28], and data analyzed using two-way ANOVA and Dunnet’s post-hoc test.

### Statistical analysis

Statistical analyses were performed using GraphPad Prism software version 9. Data were log-transformed before analysis, but plotted data are geometric means. Samples with no detectable CFUs were assigned a value of 1 CFU, to enable transformation and statistical analysis.

## Supporting information

S1 Appendix Fig A. EHEC strain growth and degradation by the paramecia vector. (A) Paramecia were incubated with EHEC to initiate bacterial uptake, external EHEC removed by extensive washing, and bacterial burden per paramecia determined from 0-6 hours post loading. At each time point, EHEC CFUs were determined by dilution plating, and number of viable and total paramecia determined using a cell counter. CFU/paramecia was calculated and data are means and SEM from N = 3 independent experiments. (B) Growth of EHEC strains in LB was determined by measuring OD600 every hour using a FluoStarOmega plate reader. Throughout the experiment, cultures were incubated at 37°C at 120 rpm. Data are means and SEM from N = 3 independent experiments. Fig B. Stx2 phage does not lyse key members of the fish microbiota. Phage plaque assays on lawns of *E. coli* MG1655 (A), *Pseudomonas* isolates zfem001–005 from the zebrafish intestine (B-F) as a host. Cultures of EHEC strains EDL933 or 86-24 were induced with mitomycin C, and sterile filtered supernatants spotted on plates containing top agar with host strains as indicated. Cell-free LB + MMC was used as a negative control. Fig C. The presence of recombinant, purified Stx2 does not affect bacterial growth. Growth of EHEC 86-24 (A), or *Pseudomonas* strains zfem001–005 isolated from the zebrafish gut (B-F) in LB containing 1 µg/ml Stx2 (purple) or an equivalent volume of E3 medium only (pink) was determined by measuring OD600 every 30 minutes using a FluoStarOmega plate reader. Throughout the experiment, cultures were incubated at 37°C at 120 rpm. Data are means and SEM from N = 3 independent experiments. Fig D. Growth of *Pseudomonas* gut isolates in not changed by components of the shed media. *Pseudomonas* strains zfem001–005 (A-D) were diluted to an OD600 of 0.1 in a 1:1 mixture of LB and either fresh E3 (pink), or sterile filtered E3 recovered from wells containing fish treated with Stx2 for 24 hours (shed media, black). Throughout the experiment, cultures were incubated at 37°C at 120 rpm and OD600 measured every 2 hrs. Data are means and SEM from N = 3 independent experiments. Fig E. Shiga toxin 2 accelerates intestinal transit. 7 dpf fish were fed fluorescent tracer food and immersed in vehicle control (untreated, A), 100 ng/mL (B) or 1000 ng/mL WT Stx2 (C), or 1000 ng/ml Stx2 mutant (D), and tracer location was scored at 3, 6, 8, and 24 hpi (black, pink, green, purple, respectively). Data are number of larvae per transit zone over time (N = 150 fish/group over three independent experiments); zone 5 corresponds to empty gut. For statistical analysis, see Fig 6G. Fig F. Shiga toxin 2 and caffeine both accelerate intestinal transit. For gut transit assays, fish were fed fluorescent tracer food and immersed in vehicle control, 200 µM or 1 mM caffeine, 100–1000 ng/mL WT Stx2, or 1000 ng/ml Stx2 mutant, and tracer location was scored at 2, 4, 6, and 24 hpi. Data are means ± sem from N = 2 independent experiments (N = 10 fish/group); Statistics: two-way ANOVA and Dunnet’s multiple comparisons test. ****p ≤ 0.0001, ***p ≤ 0.001, **p ≤ 0.01, *p < 0.05, ns (not significant) p ≥ 0.05. Fig G. Shiga toxin 2 and caffeine both facilitate displacement of endogenous *Pseudomonas ssp.* from the intestine. *Pseudomonas* CFUs recovered from larval tissue homogenates (A) or shed into the fish media (B) at 7 dpf following immersion of larval zebrafish in E3 + buffer control (untreated), 200 µM or 1 mM caffeine, 100–1000 ng/mL WT Stx2, or 1000 ng/ml Stx2 mutant for 24 hours. Data are individual fish (N = 15) or media samples (N = 10), and means ± sem over 3 independent experiments. Statistics: ANOVA and Dunnett’s multiple comparison test, ****p ≤ 0.0001, **p ≤ 0.01, *p < 0.05, ns (not significant) p ≥ 0.05; Samples with no detectable bacterial burden were plotted as 1 for visualization and statistical analysis. Dashed line = limit of detection. Fig H. Diversity and community structure analyses of the zebrafish gut microbiome across infection and Stx2 intoxication conditions. (A) Alpha diversity across experimental categories. Four diversity metrics (y-axis; Shannon, Simpson, Observed richness, Chao1) plotted as a function of four experimental categories: Controls, EHEC Infection, Genetic Rescue (Δstx2::stx2-complemented strain), and Stx2 Intoxication. Box fill colors represent experimental category; individual points represent samples colored by experimental group as shown in the legend. Boxes show median and interquartile range; whiskers extend to 1.5 × IQR. Pairwise Wilcoxon rank-sum comparisons between categories shown; all comparisons non-significant (Kruskal-Wallis: Shannon p = 0.909, Observed p = 0.720, Chao1 p = 0.632, Simpson p = 0.757). (B) CLR-PCA based on Aitchison distance (PS1, n = 72). All 8 experimental groups PERMANOVA R² = 0.143, p = 0.002. (C) Four experimental categories (Controls, EHEC Infection, Genetic Rescue, Stx2 Intoxication) PERMANOVA R² = 0.062, p = 0.001. (D) Within EHEC infection groups only (Stx-Mutant, 86-24) PERMANOVA R² = 0.081, p = 0.569. Diamonds = group centroids; 95% confidence ellipses shown. (E) Differential abundance: Welch t-test on CLR-transformed values vs Paramecium (PS1). Heatmap shows −log10 (raw p-value) for 21 core genera across 7 group comparisons. Brighter red = stronger signal. BH correction applied across all 147 tests. + = uncorrected trend (p_raw < 0.05). No genera survived BH correction. Color threshold: − log10(0.05) = 1.3. Table A. Plasmids used in this study.
(DOCX)
