# Supplementary material for: Shiga toxin increases intestinal transit to displace resident microbes and facilitate pathogen colonization
Source: PLoS Pathog. 2026 Mar 30;22(3):e1014104. doi: 10.1371/journal.ppat.1014104 (PMC13048473; doi:10.1371/journal.ppat.1014104)
Supplement: S1 Appendix — Fig A. EHEC strain growth and degradation by the paramecia vector. (A) Paramecia were incubated with EHEC to initiate bacterial uptake, external EHEC removed by extensive washing, and bacterial burden per paramecia determined from 0-6 hours post loading. At each time point, EHEC CFUs were determined by dilution plating, and number of viable and total paramecia determined using a cell counter. CFU/paramecia was calculated and data are means and SEM from N = 3 independent experiments. (B) Growth of EHEC strains in LB was determined by measuring OD600 every hour using a FluoStarOmega plate reader. Throughout the experiment, cultures were incubated at 37°C at 120 rpm. Data are means and SEM from N = 3 independent experiments. Fig B. Stx2 phage does not lyse key members of the fish microbiota. Phage plaque assays on lawns of E. coli MG1655 (A), Pseudomonas isolates zfem001–005 from the zebrafish intestine (B-F) as a host. Cultures of EHEC strains EDL933 or 86-24 were induced with mitomycin C, and sterile filtered supernatants spotted on plates containing top agar with host strains as indicated. Cell-free LB + MMC was used as a negative control. Fig C. The presence of recombinant, purified Stx2 does not affect bacterial growth. Growth of EHEC 86-24 (A), or Pseudomonas strains zfem001–005 isolated from the zebrafish gut (B-F) in LB containing 1 µg/ml Stx2 (purple) or an equivalent volume of E3 medium only (pink) was determined by measuring OD600 every 30 minutes using a FluoStarOmega plate reader. Throughout the experiment, cultures were incubated at 37°C at 120 rpm. Data are means and SEM from N = 3 independent experiments. Fig D. Growth of Pseudomonas gut isolates in not changed by components of the shed media. Pseudomonas strains zfem001–005 (A-D) were diluted to an OD600 of 0.1 in a 1:1 mixture of LB and either fresh E3 (pink), or sterile filtered E3 recovered from wells containing fish treated with Stx2 for 24 hours (shed media, black). Throughout the experiment, cult [file ppat.1014104.s001.docx]

Supplementary Information for

**Shiga toxin increases intestinal transit to displace resident microbes and facilitate pathogen colonization**

Max A. Odem^1, #, $^, Sabona B. Simbassa^1, 2, $^, Cecilia Fadhel Alvarez^1, 2, $^, Oliyad Jeilu^1, $^, Shelby R. Simar^3^, Rachel Bosserman^1^, Soumita Dutta^1^, Walter Galdamez^1, 2^, Hossaena Ayele^3^, Blake M. Hanson^3^, Diana M. Proctor^1, 2^, Anne Marie Krachler^1, 2 *^

^1^Department of Microbiology and Molecular Genetics, The University of Texas Health Science Center at Houston, Houston, Texas, USA

^2^Microbiology and Infectious Diseases Program, University of Texas MD Anderson Cancer Center UTHealth Houston Graduate School of Biomedical Sciences, Houston, Texas, USA

^3^Center for Infectious Diseases, The UTHealth Houston School of Public Health at Houston, Houston, Texas, USA

^#^Current address: Department of Neuroscience, Baylor College of Medicine, Houston, Texas, USA

^$^These authors contributed equally to the manuscript.

^*^[anne.marie.krachler@uth.tmc.edu](mailto:anne.marie.krachler@uth.tmc.edu)

**This file includes:**

S1 Appendix Figs A-H

S1 Appendix Table A

Supporting References


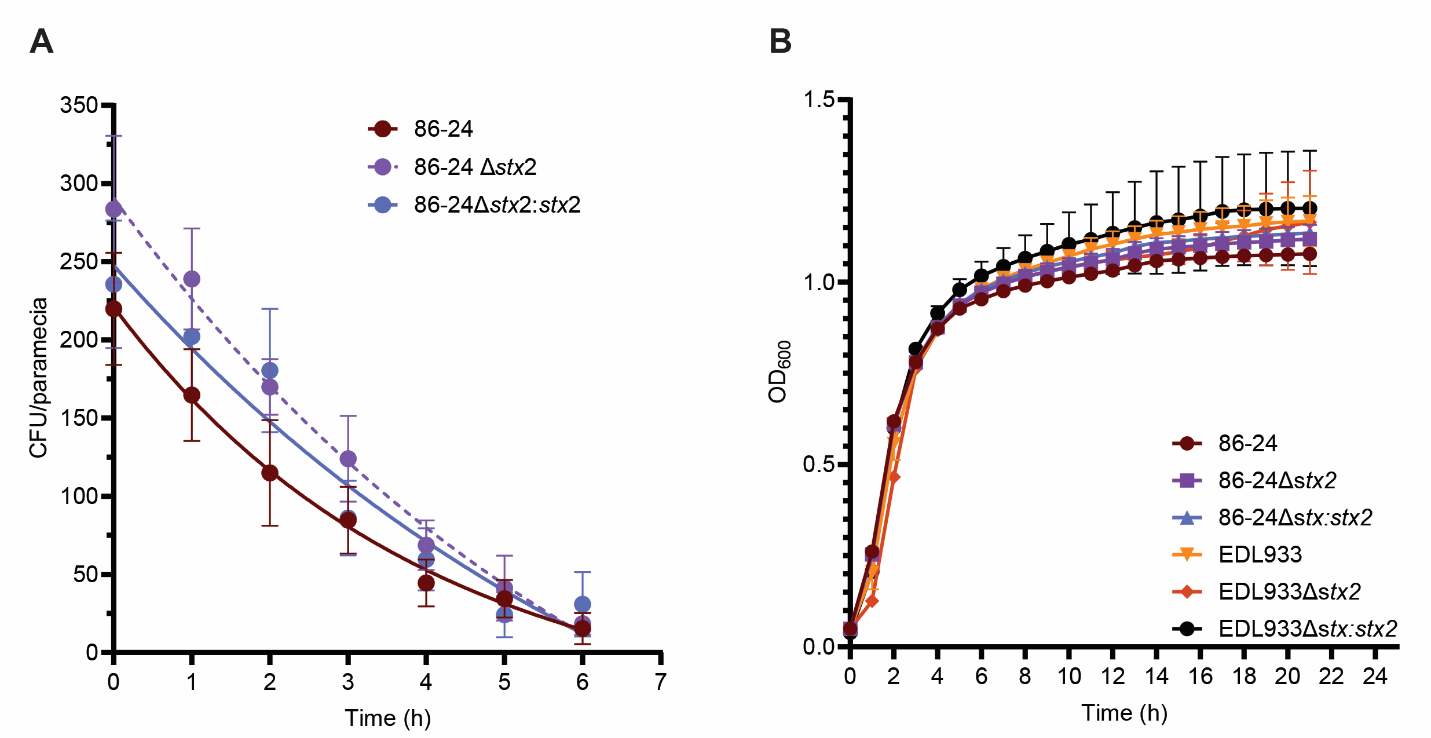


**Fig A. EHEC strain growth and degradation by the paramecia vector. (A)** Paramecia were incubated with EHEC to initiate bacterial uptake, external EHEC removed by extensive washing, and bacterial burden per paramecia determined from 0-6 hours post loading. At each time point, EHEC CFUs were determined by dilution plating, and number of viable and total paramecia determined using a cell counter. CFU/paramecia was calculated and data are means and SEM from N=3 independent experiments. **(B)** Growth of EHEC strains in LB was determined by measuring OD_600_ every hour using a FluoStarOmega plate reader. Throughout the experiment, cultures were incubated at 37 °C at 120 rpm. Data are means and SEM from N=3 independent experiments.

**
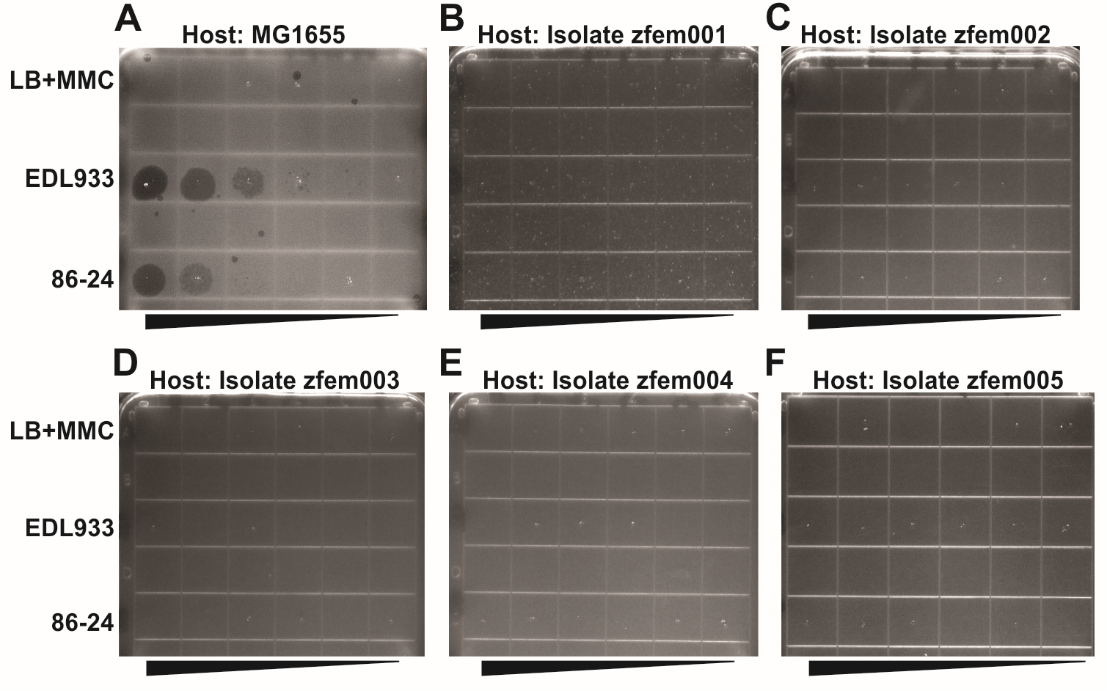
**

**Fig B. Stx2 phage does not lyse key members of the fish microbiota.** Phage plaque assays on lawns of *E. coli* MG1655 **(A)**, *Pseudomonas* isolates zfem001-005 from the zebrafish intestine **(B-F)** as a host. Cultures of EHEC strains EDL933 or 86-24 were induced with mitomycin C, and sterile filtered supernatants spotted on plates containing top agar with host strains as indicated. Cell-free LB + MMC was used as a negative control.


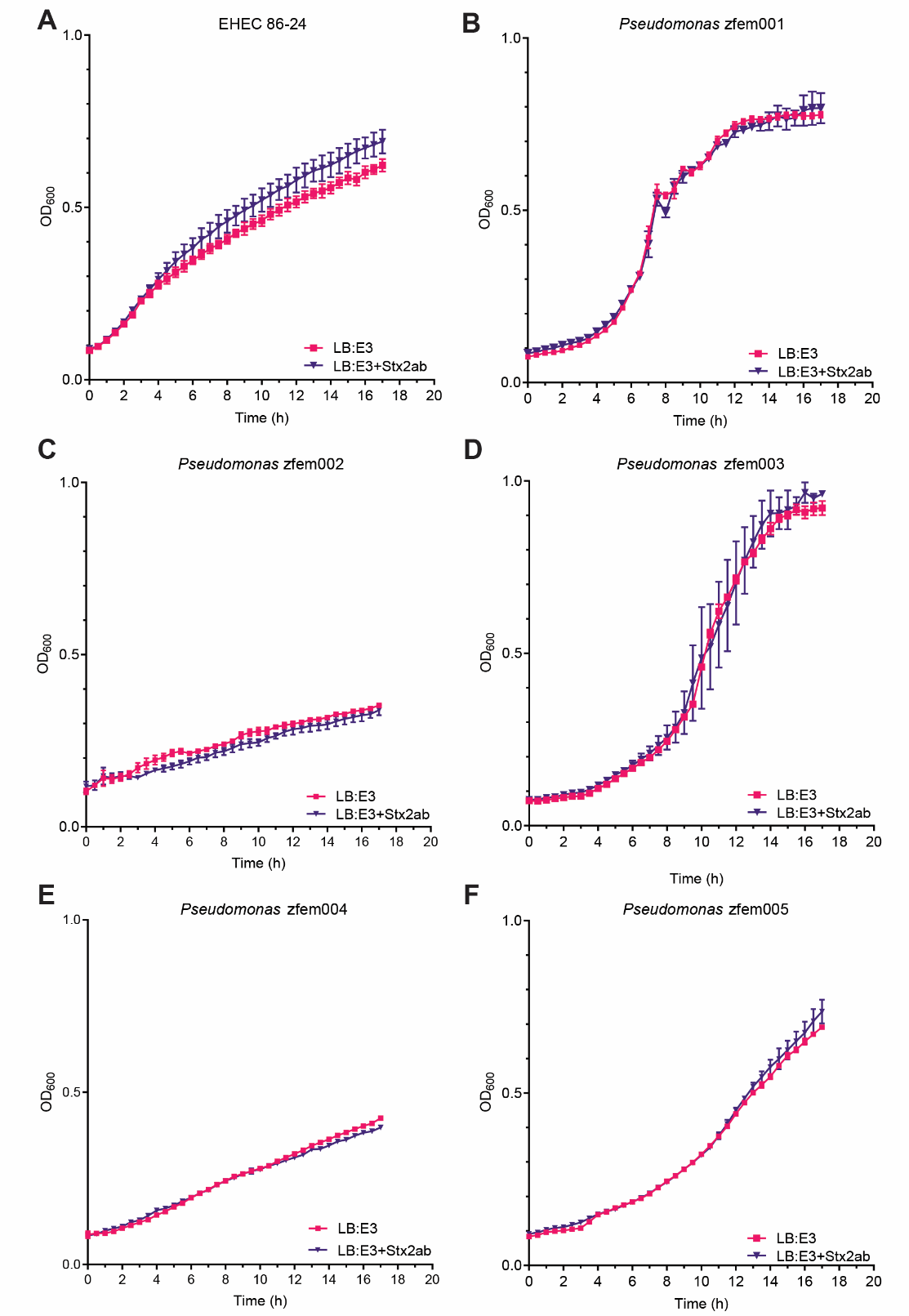


**Fig C. The presence of recombinant, purified Stx2 does not affect bacterial growth.** Growth of EHEC 86-24 **(A)**, or *Pseudomonas* strains zfem001-005 isolated from the zebrafish gut **(B-F)** in LB containing 1 µg/ml Stx2 (purple) or an equivalent volume of E3 medium only (pink) was determined by measuring OD_600_ every 30 minutes using a FluoStarOmega plate reader. Throughout the experiment, cultures were incubated at 37 °C at 120 rpm. Data are means and SEM from N=3 independent experiments.


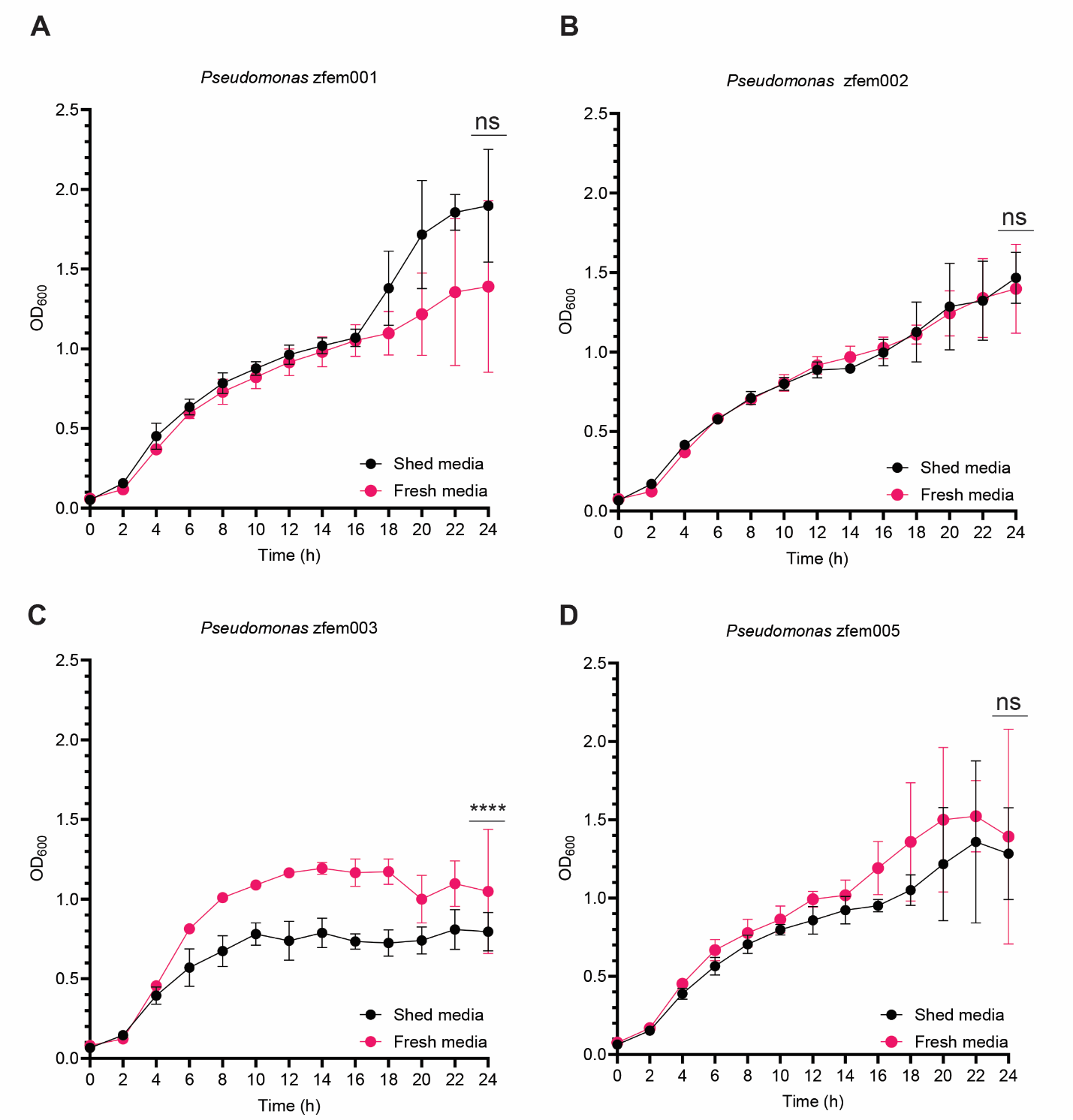


**Fig D. Growth of *Pseudomonas* gut isolates in not changed by components of the shed media.** *Pseudomonas* strains zfem001-005 **(A-D)** were diluted to an OD600 of 0.1 in a 1:1 mixture of LB and either fresh E3 (pink), or sterile filtered E3 recovered from wells containing fish treated with Stx2 for 24 hours (shed media, black). Throughout the experiment, cultures were incubated at 37 °C at 120 rpm and OD_600_ measured every 2 hrs. Data are means and SEM from N=3 independent experiments.

**
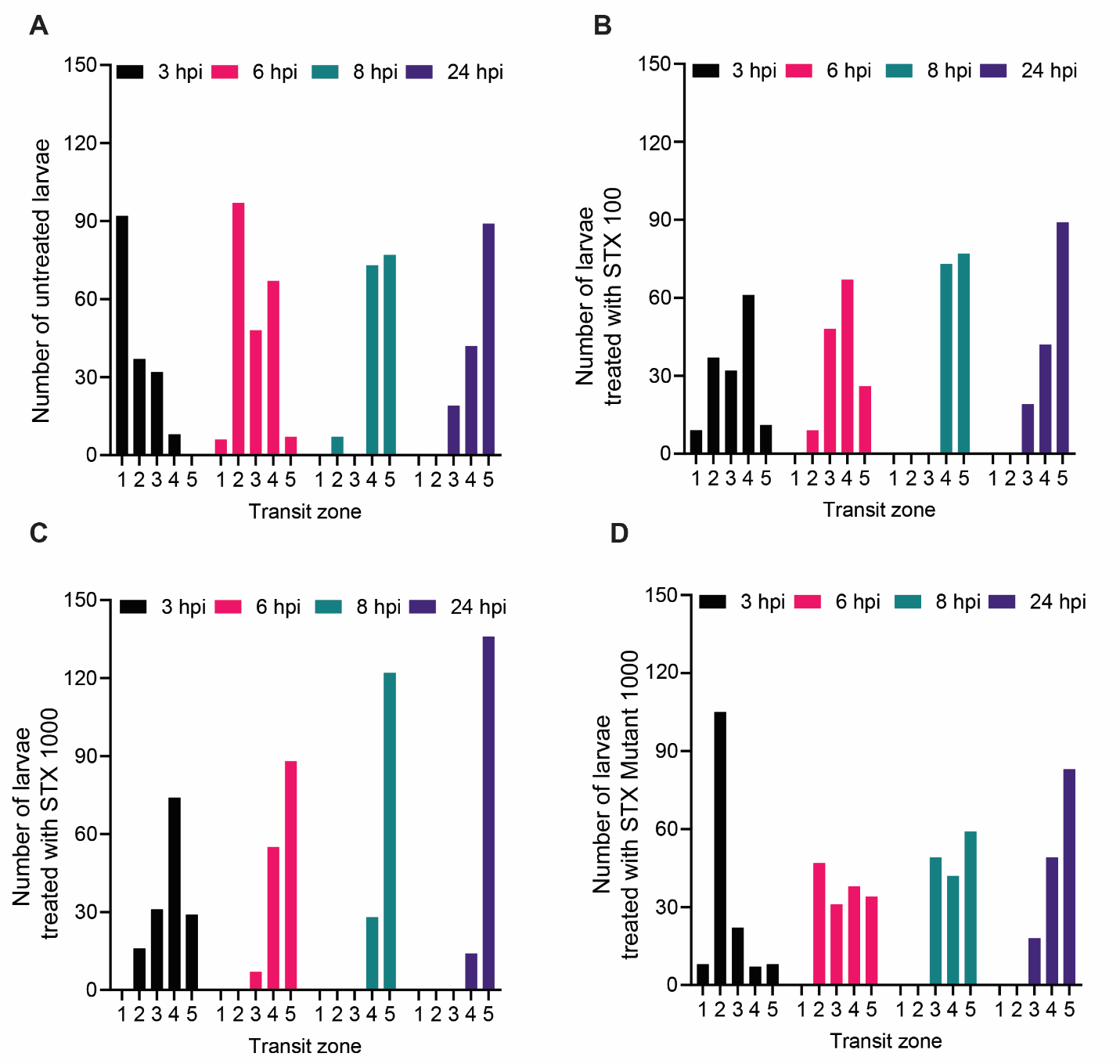
**

**Fig E. Shiga toxin 2 accelerates intestinal transit.** 7 dpf fish were fed fluorescent tracer food and immersed in vehicle control (untreated, **A**), 100 ng/mL **(B)** or 1000 ng/mL WT Stx2 **(C)**, or 1000 ng/ml Stx2 mutant **(D)**, and tracer location was scored at 3, 6, 8, and 24 hpi (black, pink, green, purple, respectively). Data are number of larvae per transit zone over time (N=150 fish/group over three independent experiments); zone 5 corresponds to empty gut. For statistical analysis, see Fig. 6G.

**
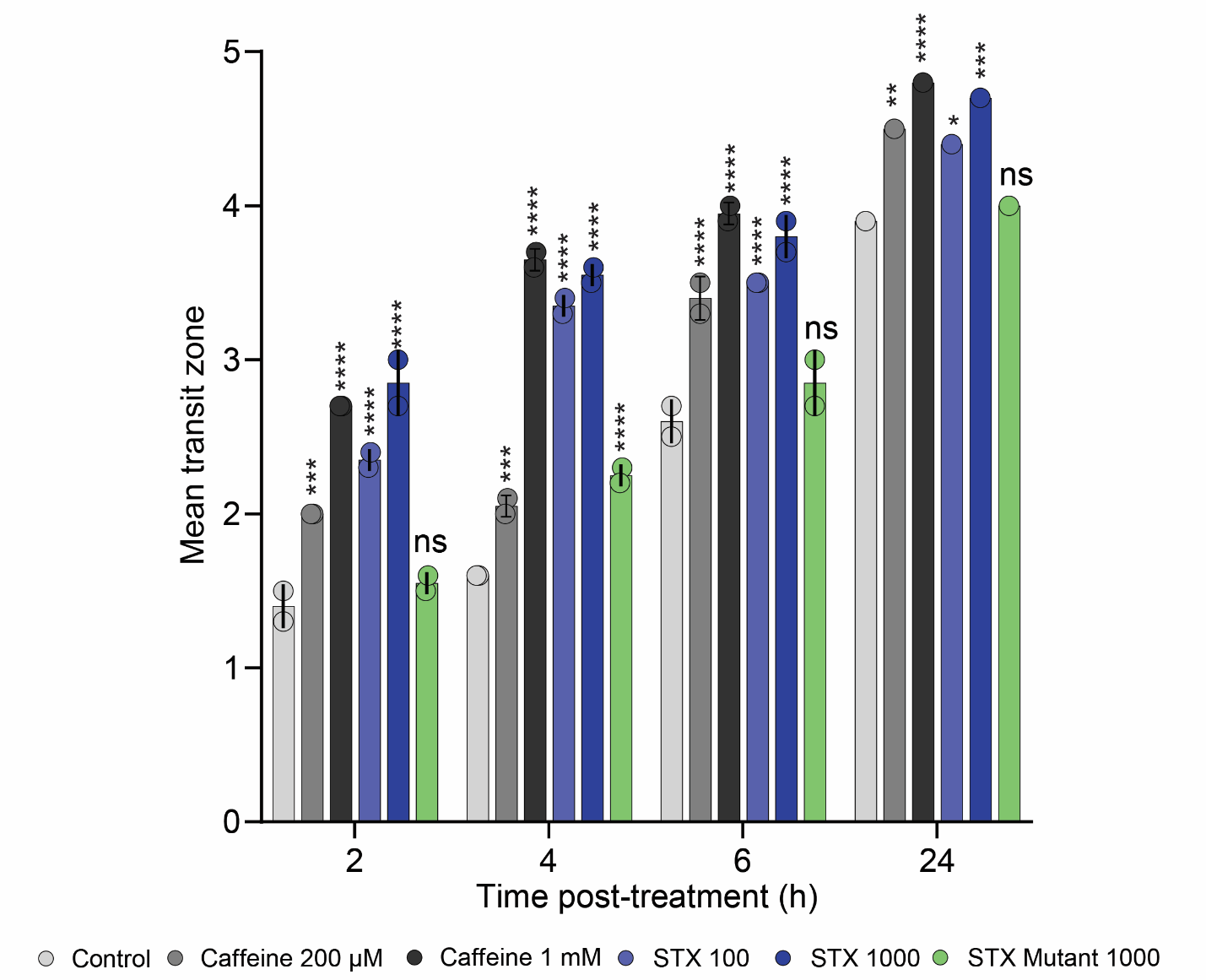
**

**Fig F. Shiga toxin 2 and caffeine both accelerate intestinal transit.** For gut transit assays, fish were fed fluorescent tracer food and immersed in vehicle control, 200 µM or 1 mM caffeine, 100-1000 ng/mL WT Stx2, or 1000 ng/ml Stx2 mutant, and tracer location was scored at 2, 4, 6, and 24 hpi. Data are means ± sem from N=2 independent experiments (N=10 fish/group); Statistics: two-way ANOVA and Dunnet’s multiple comparisons test. ****p≤0.0001, ***p≤0.001, **p≤0.01, *p<0.05, ns (not significant) p≥0.05

**
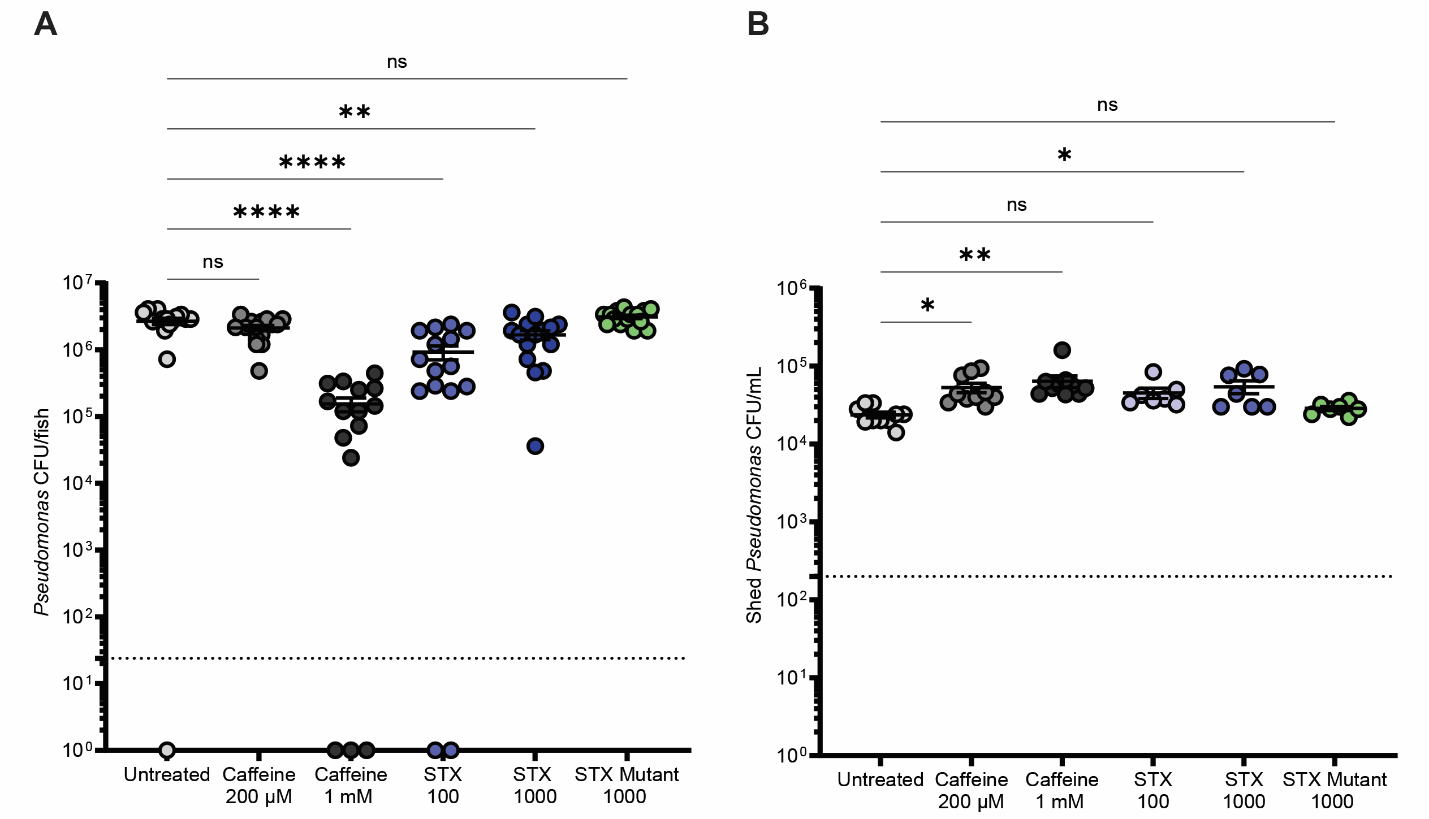
**

**Fig G. Shiga toxin 2 and caffeine both facilitate displacement of endogenous *Pseudomonas ssp.* from the intestine.** *Pseudomonas* CFUs recovered from larval tissue homogenates **(A)** or shed into the fish media **(B)** at 7 dpf following immersion of larval zebrafish in E3 + buffer control (untreated), 200 µM or 1 mM caffeine, 100-1000 ng/mL WT Stx2, or 1000 ng/ml Stx2 mutant for 24 hours. Data are individual fish (N=15) or media samples (N=10), and means ± sem over 3 independent experiments. Statistics: ANOVA and Dunnett’s multiple comparison test, ****p≤0.0001, **p≤0.01, *p<0.05, ns (not significant) p≥0.05; Samples with no detectable bacterial burden were plotted as 1 for visualization and statistical analysis. Dashed line = limit of detection.

**
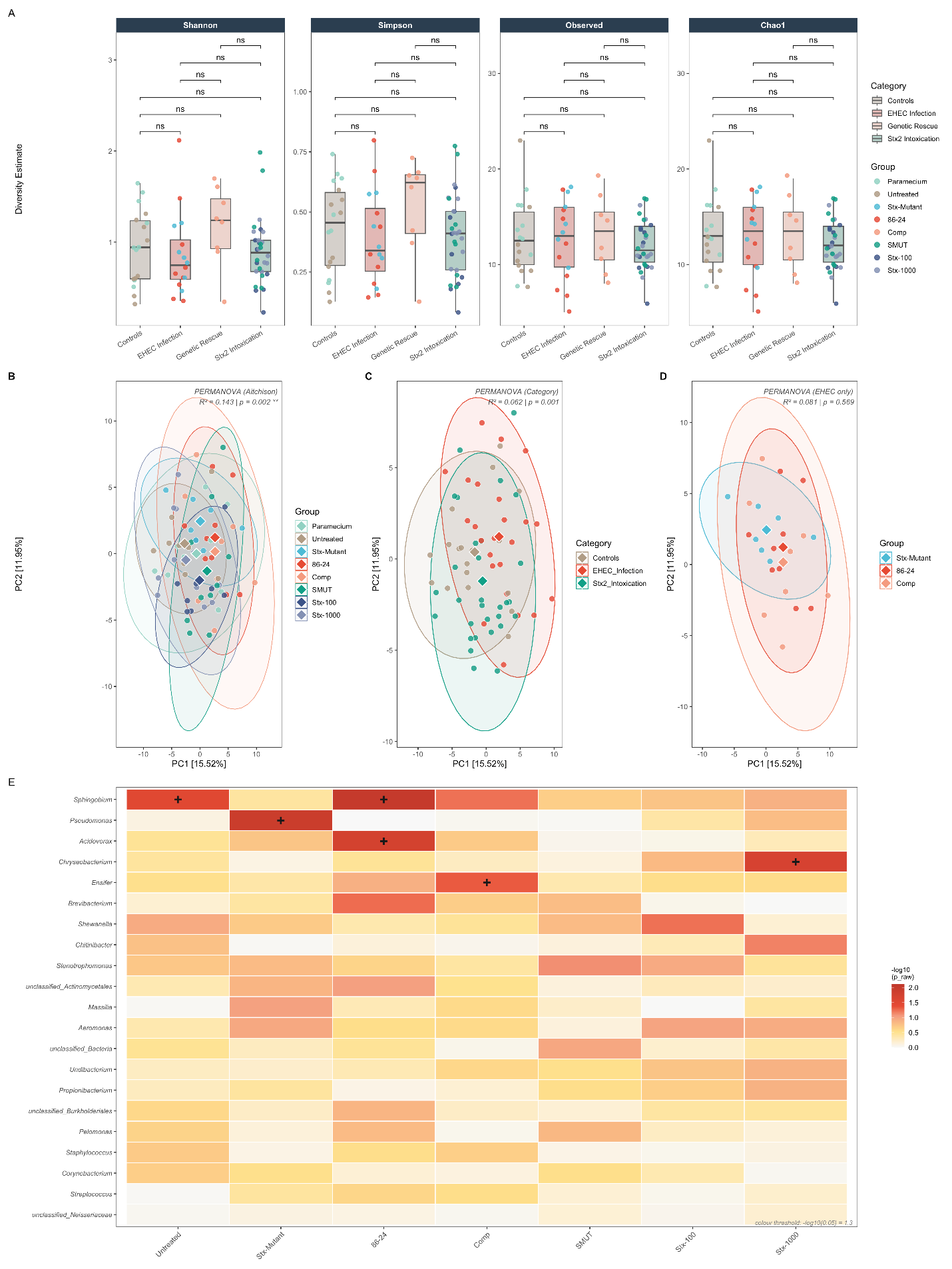
**

**Fig H. Diversity and community structure analyses of the zebrafish gut microbiome across infection and Stx2 intoxication conditions. (A) Alpha diversity across experimental categories.** Four diversity metrics (y-axis; Shannon, Simpson, Observed richness, Chao1) plotted as a function of four experimental categories: Controls, EHEC Infection, Genetic Rescue (Δstx2::stx2-complemented strain), and Stx2 Intoxication. Box fill colors represent experimental category; individual points represent samples colored by experimental group as shown in the legend. Boxes show median and interquartile range; whiskers extend to 1.5× IQR. Pairwise Wilcoxon rank-sum comparisons between categories shown; all comparisons non-significant (Kruskal-Wallis: Shannon p=0.909, Observed p=0.720, Chao1 p=0.632, Simpson p=0.757). **(B)** **CLR-PCA based on Aitchison distance (PS1, n=72).** All 8 experimental groups PERMANOVA R²=0.143, p=0.002. **(C)** Four experimental categories (Controls, EHEC Infection, Genetic Rescue, Stx2 Intoxication) PERMANOVA R²=0.062, p=0.001. **(D)** Within EHEC infection groups only (Stx-Mutant, 86-24) PERMANOVA R²=0.081, p=0.569. Diamonds = group centroids; 95% confidence ellipses shown. **(E) Differential abundance: Welch t-test on CLR-transformed values vs Paramecium (PS1).** Heatmap shows −log10 (raw p-value) for 21 core genera across 7 group comparisons. Brighter red = stronger signal. BH correction applied across all 147 tests. + = uncorrected trend (p_raw<0.05). No genera survived BH correction. Color threshold: −log10(0.05) = 1.3.

**Table A. Plasmids used in this study**

| **Plasmid** | **Description** | **Source** |
| --- | --- | --- |
| pDOC-K | Used as a template to amplify kan cassette; | [50] |
| pDOC-C | Recombineering plasmid; | [50] |
| pDOC-C-Δstx2b | pDOC-C containing kan cassette flanked by 60bp homology regions up- and downstream of stx2b; | This study |
| pACBSCE | Recombineering plasmid, ara inducible I-SceI and λ-Red expression; | [50] |
| pSTNSK-Cm | pSTNSK containing chl cassette; | This study |
| pGP-TN7-pstx2ab | pGP-TN7 containing stx2ab genes and promoter region, Gm^R^ | This study |
| pBADmychis-stx2ab | pBADmychis containing ara inducible stx2ab genes; amp^R^ | - This study |
| pBADmychis-stx2ab E189Q R192L | pBADmychis-stx2ab with Stx2B E189Q R192L mutations; amp^R^ | - This study |
| pME6032 mCherry | Plasmid for mCherry expression; tet^R^ | - [62] |

**Supporting References**

1. Lee DJ*, et al.* (2009) Gene doctoring: a method for recombineering in laboratory and pathogenic Escherichia coli strains. *BMC Microbiol* 9:252.

2. Heeb S*, et al.* (2000) Small, stable shuttle vectors based on the minimal pVS1 replicon for use in gram-negative, plant-associated bacteria. *Mol Plant Microbe Interact* 13(2):232-237.

3. Wells JG*, et al.* (1983) Laboratory investigation of hemorrhagic colitis outbreaks associated with a rare Escherichia coli serotype. *J Clin Microbiol* 18(3):512-520.

4. Tarr PI*, et al.* (1989) Genotypic variation in pathogenic Escherichia coli O157:H7 isolated from patients in Washington, 1984-1987. *J Infect Dis* 159(2):344-347.

5. Studier FW & Moffatt BA (1986) Use of bacteriophage T7 RNA polymerase to direct selective high-level expression of cloned genes. *Journal of molecular biology* 189(1):113-130.

6. Blattner FR*, et al.* (1997) The complete genome sequence of Escherichia coli K-12. *Science* 277(5331):1453-1462.

7. Simbassa SB, Clark J, Salazar K, Maresso A, & Krachler AM (2024) Draft genome sequences of Pseudomonas strains zfem001-005 isolated from the intestine of larval zebrafish Danio rerio. *Microbiol Resour Announc* 13(3):e0093423.
